# Single and Double Mutations in Tomato Ripening Transcription Factors Have Distinct Effects on Fruit Development and Quality Traits

**DOI:** 10.3389/fpls.2021.647035

**Published:** 2021-04-27

**Authors:** Jaclyn A. Adaskaveg, Christian J. Silva, Peng Huang, Barbara Blanco-Ulate

**Affiliations:** Department of Plant Sciences, University of California, Davis, Davis, CA, United States

**Keywords:** fruit ripening, fruit senescence, fruit quality, CNR, NOR, RIN, ethylene, abscisic acid

## Abstract

Spontaneous mutations associated with the tomato transcription factors COLORLESS NON-RIPENING (SPL-CNR), NON-RIPENING (NAC-NOR), and RIPENING-INHIBITOR (MADS-RIN) result in fruit that do not undergo the normal hallmarks of ripening but are phenotypically distinguishable. Here, we expanded knowledge of the physiological, molecular, and genetic impacts of the ripening mutations on fruit development beyond ripening. We demonstrated through phenotypic and transcriptome analyses that *Cnr* fruit exhibit a broad range of developmental defects before the onset of fruit ripening, but fruit still undergo some ripening changes similar to wild type. Thus, *Cnr* should be considered as a fruit developmental mutant and not just a ripening mutant. Additionally, we showed that some ripening processes occur during senescence in the *nor* and *rin* mutant fruit, indicating that while some ripening processes are inhibited in these mutants, others are merely delayed. Through gene expression analysis and direct measurement of hormones, we found that *Cnr*, *nor*, and *rin* have alterations in the metabolism and signaling of plant hormones. *Cnr* mutants produce more than basal levels of ethylene, while *nor* and *rin* accumulate high concentrations of abscisic acid. To determine genetic interactions between the mutations, we created for the first time homozygous double mutants. Phenotypic analyses of the double ripening mutants revealed that *Cnr* has a strong influence on fruit traits and that combining *nor* and *rin* leads to an intermediate ripening mutant phenotype. However, we found that the genetic interactions between the mutations are more complex than anticipated, as the *Cnr/nor* double mutant fruit has a *Cnr* phenotype but displayed inhibition of ripening-related gene expression just like *nor* fruit. Our reevaluation of the *Cnr*, *nor*, and *rin* mutants provides new insights into the utilization of the mutants for studying fruit development and their implications in breeding for tomato fruit quality.

## Introduction

Fleshy fruit gain most of their quality traits, such as color, texture, flavor, and nutritional value, as a result of physiological and biochemical changes associated with ripening. Fruit ripening has been studied for decades, yet there are still many unanswered questions about the timing and coordination of the biological processes related to this developmental program. Much of this research has been done in the model for fleshy fruit ripening, tomato (*Solanum lycopersicum*), and has utilized the spontaneous single ripening mutants *Cnr* (*Colorless non-ripening*), *nor (non-ripening)*, and *rin (ripening inhibitor)* (Robinson and Tomes, 1968; Tigchelaar et al., 1973; Thompson et al., 1999; Giovannoni et al., 2004; Manning et al., 2006). Each of these mutations produces pleiotropic defects to ripening and occur in or near genes encoding the transcription factors (TFs) SPL-CNR, NAC-NOR, and MADS-RIN, belonging to the SQUAMOSA promoter binding protein-like (SPL), NAM, ATAF1/2, CUC2 (NAC) and, MCM1, AG, DEF, SRF (MADS) TF families, respectively. Each TF family functions in diverse developmental processes and have distinct spatiotemporal expression patterns (Karlova et al., 2014; Shinozaki et al., 2018).

These mutants were used to study ripening under the assumption that the mutations cause a complete loss of function to the corresponding protein. Recently, it has been discovered that the *nor* and *rin* mutations produce proteins that are still functional and gain the ability to negatively regulate their targets (Ito et al., 2017; Li et al., 2018, 2019b; Gao et al., 2019, 2020; Wang et al., 2019). In *nor*, the two base pair deletion truncates the protein but still produces a functional DNA-binding and dimerizing NAC domain (Gao et al., 2020). In *rin*, a large deletion creates a chimeric protein with the neighboring gene *MACROCALYX* (*MC*), producing a functional protein with suppression activity (Ito et al., 2017). The *Cnr* mutation is also thought to be a gain of function mutation, although the mechanism has yet to be understood (Gao et al., 2019). The *Cnr* mutation results from hypermethylation upstream of the gene near the promoter and has been shown to inhibit the genome-wide demethylation cascade associated with normal tomato ripening (Zhong et al., 2013). Previously, these TFs were regarded as master regulators of ripening; however, given the new information about the nature of the mutations in *Cnr*, *nor*, and *rin*, it is less clear the precise roles the TFs are playing in ripening (Giovannoni et al., 2017; Wang et al., 2020a).

The *nor* and *rin* mutants have been utilized in breeding for developing tomato hybrids with extended shelf life or extended field harvest depending on their purpose for the fresh market and processing tomato industries (Kopeliovitch et al., 1979; Kitagawa et al., 2005; Garg et al., 2008; Osei et al., 2017). Hybrids between elite varieties and the ripening mutants have a delayed ripening progression, but with the tradeoff of decreased fruit quality attributes, such as color, taste, and aroma (Kitagawa et al., 2005; Tieman et al., 2017). Although there are some publications dedicated to evaluating the physiological characteristics of mutant or hybrid fruit (Tigchelaar et al., 1978; Agar et al., 1994; Garg et al., 2008), up to this point, much of what we know about the ripening mutations is based on controlled greenhouse experiments with limited fruit and few ripening stages examined. A complete dataset of phenotypic data produced from large-scale field trials evaluating fruit ripening and senescence is lacking to provide information relevant to breeding, particularly in the new context of the molecular mechanisms behind the *nor* and *rin* mutations.

The *Cnr* mutant provides a unique opportunity to study the role of epigenetics in fruit ripening but is not used in breeding because the mutant phenotype is dominant. *Cnr* has been regarded as a ripening mutant due to its unique colorless phenotype and additional ripening defects (Thompson et al., 1999). It has been suggested that *Cnr* fruit undergo normal growth and development (Lai et al., 2020); however, fruit appear different from wild type (WT) even before ripening, with a smaller size, alterations in cell wall enzyme expression, and earlier chlorophyll degradation (Eriksson et al., 2004; Wang et al., 2020b). To better utilize *Cnr* as a tool for studying fruit development and ripening, a broader understanding of the physiological and transcriptomic alterations in this mutant is necessary.

These spontaneous single mutants need to be reevaluated as tools to understand the wide-ranging biological processes regulated by each TF. Previous literature has generally assumed that the mutations block ripening, resulting in similar processes affected (Giovannoni, 2007; Karlova et al., 2014; Giovannoni et al., 2017; Osorio et al., 2020). This study demonstrates that each mutant has a unique ripening phenotype, resulting from a combination of inhibited and delayed developmental processes. We integrated phenotypic data with gene expression data and hormone measurements in the *Cnr, nor*, and *rin* mutants across ripening and senescence to characterize the extent and timing of the ripening defects. Tomatoes grown under field conditions were assessed for fruit traits over multiple seasons. We then performed a transcriptomic analysis to gain more definition of the timing in which mutant fruit deviated from WT in their development and to determine specific molecular functions altered in each mutant. Due to their pivotal role in regulating ripening, we focused on defects in hormone networks, including biosynthesis and accumulation. We analyzed the influence of each mutation on the expression of the other TF throughout ripening and senescence. Finally, to better understand the combined genetic effects of the mutants on fruit ripening, we generated homozygous double mutants of *Cnr*, *nor*, and *rin* and used phenotyping and transcriptional data to evaluate the relationships between the mutants.

## Materials and Methods

### Plant Material

Tomato plants (*Solanum lycopersicum)* of *c.v.* ‘Alisa Craig’ and the isogenic ripening mutants *Cnr, nor*, and *rin* were grown in randomized plots under standard field conditions in Davis, CA, United States, during the 2016, 2017, 2018, and 2020 seasons. Fruit tagged at 10 days post-anthesis (dpa), which corresponds to 7 mm in fruit diameter, were harvested at stages equivalent to the WT fruit. Fruit were sampled at the mature green (MG), turning (T), red ripe (RR), and overripe (OR) stages, corresponding to 37, 45, 50, and 57 dpa, respectively. The term “RR” is used throughout the manuscript to refer to the 50 dpa stage of all genotypes, even when the mutant fruit do not turn red. Fruit stages for each of the mutants were further validated by external color analysis (see details on fruit trait phenotyping).

Double mutant fruit were generated through reciprocal crosses: *Cnr* × *nor*, *nor* × *Cnr, Cnr* × *rin*, *rin* × *Cnr, nor* × *rin*, and *rin* × *nor*. Fruit were selfed after the initial cross to generate an F2 segregating generation. The double mutants were initially selected in the F2 generation through genotyping and phenotyping. At least two additional generations after F2 were obtained through selfing to ensure the stability of the double mutations and to perform the experiments in this study. Three seasons of data were collected for the *Cnr/nor* fruit (2016, 2017, and 2020) while only one season of data was collected for the *rin/nor* and *Cnr/rin* crosses.

### Mutant Genotyping

The mutant lines were genotyped for their respective mutations. For *nor*, the Phire Plant Direct PCR Kit (Thermo Fisher Scientific, United States) was used to extract DNA and amplify the region of the gene containing the 2 bp mutation using the primers listed in Supplementary Table 1. The PCRs were run on a SimpliAmp Thermal Cycler (Applied Biosystems, United States) with the following conditions denaturation: 99°C for 5 min; 35 cycles of 98°C for 5 s, 56°C for 25 s, and 72°C for 25 s; with a final extension of 72°C for 1 min. The PCR products were purified using Wizard SV Gel and PCR Clean-Up System (Promega, United States) and then sequenced with Sanger technology to confirm the absence of the two (AA) nucleotides. For *rin*, the Phire Plant Direct PCR Kit (Thermo Fisher Scientific, United States) was used to extract DNA and perform end-point PCRs using primers specific for the mutant and WT alleles (Supplementary Table 1). The following PCR conditions were used for the WT allele primers: denaturation 99°C for 5 min; 35 cycles of 98°C for 5 s, 55°C for 25 s, and 72°C for 25 s; with a final extension of 72°C for 1 min. The PCR conditions for the mutant allele primers were: denaturation 98°C for 5 min; 40 cycles of 98°C for 5 s, 58°C for 25 s, and 72°C for 25 s; with a final extension of 72°C for 1 min. The PCR products were visualized as bands using a 1% agarose gel.

The *Cnr* epimutation was genotyped by bisulfite sequencing. Extracted DNA was treated with the Zymo Gold bisulfite kit (Zymo Research, United States). Bisulfite treated-DNA was PCR amplified for the *CNR* promoter region containing the methylation changes (Manning et al., 2006) using the primers listed in Supplementary Table 1. The following PCR conditions were used: 94°C for 2 min; 40 cycles of 94°C for 30 s, 54°C for 30 s, and 60°C for 45 s, and a final extension of 60°C for 10 min. The PCR products were then Sanger sequenced and compared to the same region amplified in untreated controls with primers (Supplementary Table 1). The following conditions were used to amplify the untreated DNA: 95°C for 2 min; 35 cycles of 95°C for 30 s, 56°C for 30 s and 72°C for 1 min, and a final extension of 72°C for 10 min. To ensure mutants were homozygous for the locus, we confirmed the double mutants by allowing the plants to self for at least two additional generations and checking that the progeny were not segregating for any fruit phenotypes.

### Fruit Trait Phenotyping

Fruit trait data were collected across four field seasons (2016, 2017, 2018, and 2020). The genotypes, developmental stages, number of biological replicates, and number of field seasons used for fruit trait phenotyping can be found in Supplementary Table 2. One season of phenotyping was performed for *Cnr/rin* and *rin/nor* double mutant fruits for color, firmness, and ethylene. Three seasons of data were collected for the *Cnr/nor* double mutant fruit for ethylene and two seasons of data for color and firmness. Fruit were collected from multiple plots or harvests to capture environmental variability. Fruit trait measurements were taken on the same day of harvest for all samples unless noted. Intact and halved fruit were imaged using the VideometerLab 3 (Videometer, Denmark) facilitated by Aginnovation LLC^1^. External color measurements were obtained from individual fruit with the CR-410 Chroma Meter (Konica Minolta Inc, Japan) and recorded in the L^∗^a^∗^b^∗^ color space, where L^∗^ quantifies lightness, a^∗^ quantifies green/red color, and b^∗^ quantifies blue/yellow color. Principal component analysis (PCA) of the color parameters was performed with the *FactoMineR* package and graphed with the *FactoExtra* package in R (Lê et al., 2008; Kassambara et al., 2017). Non-destructive firmness measurements were taken on the TA.XT2i Texture Analyzer (Texture Technologies, United States) using a TA-11 acrylic compression probe, a trigger force of 0.035 kg, and a test speed of 2.00 mm/sec with Exponent software (Texture Technologies Corporation, United States). Firmness values are reported as kilograms (kg) force. The size was measured by taking the largest diameter (mm) of the fruit with a handheld caliper.

Tomato juice was produced by pressing the fruit tissues with a juicer and filtering with cheesecloth to measure total soluble solids (TSS) and titratable acidity (TA). At least five biological replications of tomato juice were obtained from independent pools of 10–12 fruit from distinct plots in the field or at different harvest dates within the field season. TSS were measured as percent Brix with a Reichert AR6 Series automatic bench refractometer (Reichert Inc., United States) from the prepared juice with three technical replicates. TA was measured using the tomato juice with the TitraLab TIM850 Titration Manager (Radiometer Analytics, Germany). Four grams of juice were diluted with water in 20 mL of deionized water to measure TA based on citric acid equivalents. Significant differences in fruit traits across genotypes and ripening stages were determined in R (R foundation for Statistical Computing) using Type I analysis of variance (ANOVA) tests, followed by a *post hoc* test (Tukey Honest Significant Differences, HSD) using the R package *agricolae* (De et al., 2017).

### RNA Extraction

On the day of harvest, the fruit pericarp tissues were dissected and flash-frozen in liquid nitrogen. Frozen tissues were then ground to a fine powder with the Retsch Mixer Mill MM 400 (Verder Scientific, Netherlands). One gram of ground tissue was used for RNA extractions as described in Blanco-Ulate et al. (2013). RNA concentrations were quantified with Nanodrop One Spectrophotometer (Thermo Scientific, United States) and Qubit 3 (Invitrogen, United States). RNA integrity was then assessed on an agarose gel. Six biological replicates composed of 8–10 independent fruit were extracted per genotype and ripening stage from the 2016 and 2018 seasons.

### cDNA Preparation and RT-qPCR

cDNA was prepared from 1 μg of RNA of all samples using M-MLV Reverse Transcriptase (Promega, United States) in the SimpliAmp Thermal Cycler (Applied Biosystems, United States). RT-qPCRs were performed using PowerSYBR Green PCR Master Mix (Applied Biosystems, United States) in the QuantStudio3 (Applied Biosystems, United States) following the preset qPCR conditions for the ‘Comparative CT method.’ The tomato *SlUBQ* (*Solyc12g04474*) was used as the reference gene for all relative expression analyses. Primers for the genes of interest were designed using Primer-BLAST (Ye et al., 2012) or obtained from previous studies (Supplementary Table 1). For all new qPCR primer sets, efficiency was confirmed to be higher than 90% using fourfold DNA or cDNA dilutions (0, 1:1, 1:4, 1:16, 1:64, and 1:256) in triplicate. Then, specificity was checked by analyzing the melting curves at temperatures ranging from 60 to 95°C. Relative gene expression was calculated using the formula 2^(reference gene Ct – gene of interest Ct)^.

### cDNA Library Preparation, RNA Sequencing, and Sequencing Data Processing

Four biological replicates each of *Cnr/nor* MG and RR fruit RNA were used to prepare cDNA libraries. cDNA libraries were prepared with Illumina TruSeq RNA Sample Preparation Kit v.2 (Illumina, United States) from the extracted RNA. The quality of the barcoded cDNA libraries was assessed with the High Sensitivity DNA Analysis Kit in the Agilent 2100 Bioanalyzer (Agilent Technologies, United States) and then sequenced (50 bp single-end reads) on the Illumina HiSeq 4000 platform by the DNA Technologies Core at UC Davis Genome Center.

Raw RNAseq data from WT, *Cnr*, *nor*, and *rin* at MG and RR were obtained from a published dataset by our group (Silva et al., 2021), GEO accession GSE148217), while raw RNAseq data from the immature stages of the ripening mutants were extracted from Lü et al. (2018) (GEO accession GSE116581). The RNAseq datasets for the *Cnr/nor* double mutant were generated in this study. The raw sequencing reads from the different datasets were analyzed *de novo* following the bioinformatics pipeline described below. Raw reads were trimmed for quality and adapter sequences using Trimmomatic v0.39 (Bolger et al., 2014) with the following parameters: maximum seed mismatches = 2, palindrome clip threshold = 30, simple clip threshold = 10, minimum leading quality = 3, minimum trailing quality = 3, window size = 4, required quality = 15, and minimum length = 36. Trimmed reads were then mapped using Bowtie2 (Langmead and Salzberg, 2012) to the tomato transcriptome (SL4.0 release^2^). Count matrices were made from the Bowtie2 results using sam2counts.py v0.91^3^. A summary of all read mapping results can be found in Supplementary Table 3.

### Differential Expression Analysis, Functional Annotations, and Enrichment Analysis

The Bioconductor package *DESeq2* (Love et al., 2014) in R was used to normalize read counts and perform PCAs and differential expression analyses for various comparisons (Supplementary Tables 4, 5). Differentially expressed genes (DEGs) for each comparison had an adjusted *P*-value of less than or equal to 0.05. Gene functional annotations were retrieved from the Kyoto Encyclopedia of Genes and Genomes (KEGG) using the KEGG Automatic Annotation Server (Moriya et al., 2007). Enrichment analysis for all functional annotations was performed using a Fisher test. The *P*-values obtained from the Fisher test were adjusted with the Benjamini and Hochberg method (Benjamini and Hochberg, 1995). Shared and unique DEGs among the comparisons were determined using the R package *UpSetR* (Conway et al., 2017).

### Hormone Extraction and Analysis

Ethylene production measurements were taken from MG, RR, and OR fruit on the day of harvest. At least five biological replicates of 5–7 fruit were used for the measurements. The genotypes, developmental stages, and number of biological replicates used for ethylene analysis in each field season can be found in Supplementary Table 2. Fruit were weighed and placed in 1 L airtight glass jars. Headspace gas (3 ml) was extracted from the sealed containers after 60 min and was injected into a Shimadzu CG-8A gas chromatograph (Shimadzu Scientific Instruments, Japan). Sample peaks were measured against an ethylene standard. The rate of ethylene production (nL kg^–1^ fresh weight h^–1^) was calculated from the peak, fruit mass, and incubation time.

Frozen ground tissue prepared from the tomato fruit pericarp was lyophilized, weighed, and extracted in isopropanol:H2O:HCL1MOL(2:1:0.005) with 100 l of internal standard solution (1000 pg) as described in Casteel et al. (2015). Abscisic acid (ABA) and 1-aminocyclopropane-1-carboxylate (ACC) were measured using liquid chromatography coupled to tandem mass spectrometry and internal standards as described in Casteel et al. (2015). The hormone concentrations were expressed as ng/g of dry weight. Four to six biological replicates composed of 8–10 fruit were used for these measurements for the 2017 season. Significant differences in hormone accumulation across genotypes and ripening stages were determined using Type I ANOVA in R, followed by an HSD test using the R package *agricolae* (De et al., 2017). In some cases, pairwise comparisons in hormone accumulation were also conducted by Student’s *t-test* in R.

## Results

### Ripening Mutants Display Distinct Phenotypes and Transcriptional Profiles Throughout Fruit Development

**FIGURE 1:**
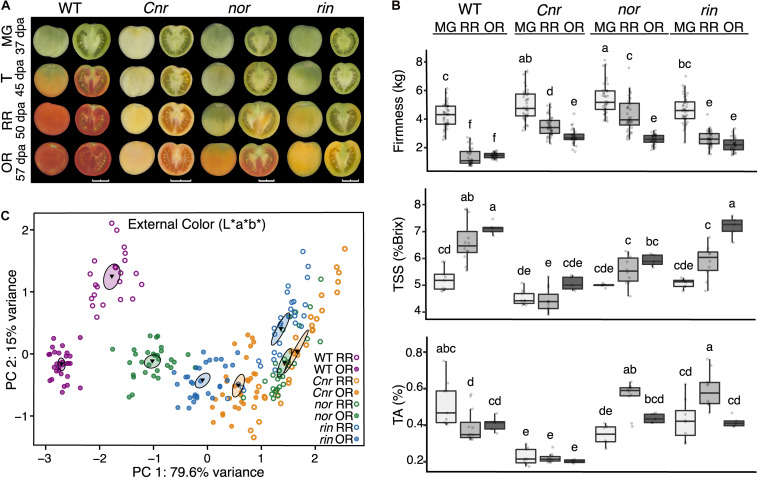
Fruit traits of the tomato single ripening mutants. **(A)** Ripening progression of wild type (WT), *c.v*. ‘Ailsa Craig,’ compared to the isogenic mutants *Cnr*, *nor*, and *rin* across four developmental stages: mature green [MG, 37 days post anthesis (dpa)], turning (T, 45 dpa), red ripe (RR, 50 dpa), and overripe (OR, 57 dpa) shown at left whole and at right in longitudinal sections. Images were captured and processed with the VideometerLab instrument. Bars correspond to 2 cm. **(B)** Measurements of fruit firmness (*n* = 28–44), total soluble solids (TSS) (*n* = 5–12), and titratable acidity (TA) (*n* = 5–12) for each MG, RR, and OR stages are presented. Error bars represent standard error between biological replicates of each sample. Letters indicate significant differences among genotypes and stages calculated by ANOVA and Tukey HSD (*P* ≤ 0.05). **(C)** Principal component analysis of external color measured on the L*a*b* color scale of each genotype at the RR (*n* = 22–34) and OR stage (*n* = 28–40). The center of gravity is represented by a triangle with surrounding ellipses indicating 95% confidence interval.

Fruit from the *Cnr*, *nor*, and *rin* mutants fail to acquire most ripening-associated traits that make them appealing for consumption. Yet, each mutant can be distinguished by their unique phenotypes (Figure 1). To determine the impact of *Cnr*, *nor*, and *rin* mutations on the key fruit traits, we measured external color, firmness, total soluble solids (TSS), and titratable acidity (TA) at multiple ripening stages. Fruit from the isogenic mutants *Cnr*, *nor*, and *rin*, were harvested alongside WT from an experimental field at selected ripening stages, mature green (MG; 37 dpa), turning (T; 45 dpa), red ripe (RR; 50 dpa), and overripe (OR; 57 dpa) (Figure 1A). We captured field variability through large sample sizes and validated across two to four independent field seasons. A summary of all seasons is displayed in Figure 1 while a breakdown of the data by field season can be found in Supplementary Table 2.

As expected, between the MG and RR stages WT fruit turned red internally and externally, reduced firmness, accumulated TSS, and became less acidic during ripening. *Cnr* fruit showed visual differences compared to all of the genotypes at the MG stage and continuing through subsequent stages, including significantly smaller size and its characteristic colorless flesh, marked by an opaque yellow coloration of the pericarp (Figure 1 and Supplementary Figure 1). Statistical analyses performed for color and size confirmed *Cnr* exhibited significant differences (*P* ≤ 0.05) consistently across each field season, as reported in Supplementary Table 2. Fruit of *nor* and *rin* displayed a distinct absence of any red coloration compared to WT at the RR stage; instead, these fruit began to turn yellow externally. Fruit of all ripening mutants were significantly firmer across all stages than the WT, though this difference was especially pronounced in *Cnr* (Figure 1B). Overall, *Cnr* was consistently different from the other genotypes before and during ripening, while *nor* and *rin* remained similar to WT MG fruit.

The OR stage was selected to investigate if the ripening mutants displayed phenotypic changes at later time points that could be associated with a delay in fruit development. At this stage, *nor* fruit started to turn orange-red externally and red internally, similar to WT fruit. The *nor* OR fruit resembled WT fruit between the T and RR stages. We performed a PCA of the color data (L*a*b measurements) to compare the genotypes at the RR and OR stage, and found *nor* OR measured closely with WT RR in external coloration (Figure 1C). A summary of the color data and statistical analyses performed can be found in Supplementary Table 2. While *nor* OR fruit visually looked most similar to WT RR fruit, *rin* OR fruit consistently measured similarly to WT fruit at the RR and OR stages in the taste-related traits of TSS and TA. These phenotypes were especially noticeable in the OR stage, suggesting that *rin* exhibits a delay in these traits. In contrast, *Cnr* remained distinct from WT and the other mutants at the OR stage in all measurements (Figure 1B). Thus, in the OR stage, *nor* and *rin* behaved more similar to WT, suggesting they display more ripening phenotypes after the RR stage.

**FIGURE 2:**
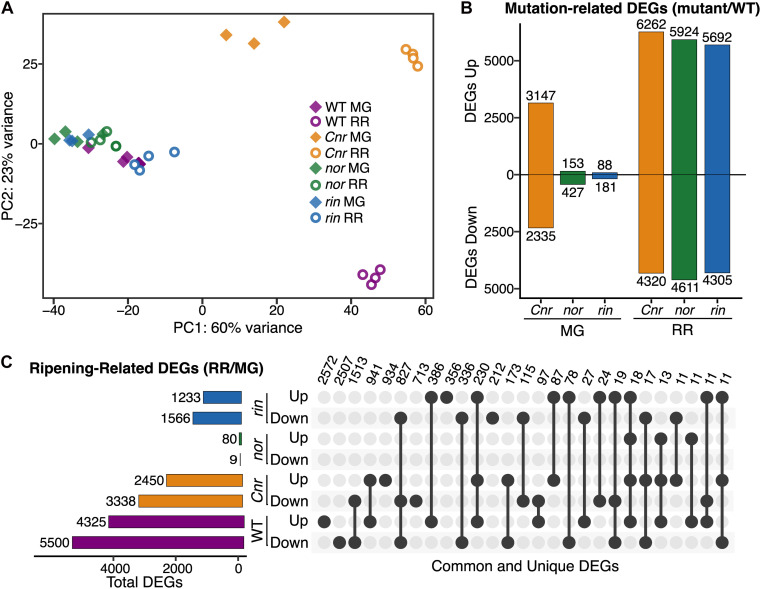
Transcriptional misregulation in fruit from the single ripening mutants *Cnr, nor*, and *rin*. **(A)** Principal component analysis of total mapped RNAseq reads for *Cnr*, *nor*, *rin* and wild type (WT) fruit at the mature green (MG) and red ripe (RR) stages. **(B)** Number of differentially expressed genes (DEGs; *P*_adj_ ≤ 0.05) up- or down-regulated for each mutant compared to WT. **(C)** Total, unique, and intersecting up- and down-regulated DEGs across ripening (RR/MG) for each genotype visualized using *UpSetR*. Dots connected by lines indicate common DEGs between categories and single dots indicate unique DEGs, with the number of genes in each category listed above.

The distinct phenotypic differences observed between the ripening mutants indicate that each mutation has a unique impact on fruit molecular processes at specific developmental stages. We performed an RNAseq study of WT, *Cnr*, *nor*, and *rin* fruit at the MG and RR stages to gain insights into the observed phenotypes. A principal component analysis (PCA) was performed using mapped normalized reads to the tomato predicted transcriptome (34,075 genes; SL4.0 release) from WT and mutant samples at MG and RR stages (Figure 2A). The PCA revealed that the genotypes were mainly separated by ripening stage (PC1, 60% variance) and that *Cnr* was distinct from WT and the other mutants (PC2, 23% variance). Remarkably, *Cnr* displayed the most similar pattern to WT across PC1 than any other mutant. Like their phenotypes suggested, *nor* and *rin* transcriptomic profiles showed little change between the MG and RR stage and clustered with the WT MG fruit. The separation driven by PC2 supported our observations that *Cnr* fruit was phenotypically different from other genotypes.

### *Cnr* Fruit Display Transcriptional Differences From Wild Type Before Ripening

Because *Cnr* showed deviation from WT at the MG stage in both phenotype and transcriptional profiles, we hypothesized that gene expression across the genome was affected prior to the MG stage. To determine when the transcriptional profile of *Cnr* began to diverge from WT and other mutants, we obtained and reanalyzed raw RNAseq data from all genotypes at four early stages of fruit growth and development (7, 17, 27, and 37 dpa) (Lü et al., 2018). We performed a PCA for each developmental stage and found that *Cnr* was separated from other genotypes as early as 7 dpa in fruit development, while *nor* and *rin* were similar to WT throughout early development (Supplementary Figure 2). When evaluating differentially expressed genes (DEGs, *P*_adj_ ≤ 0.05) between *Cnr* and WT fruit at 7 dpa, we detected 1,320 mutation-related DEGs while *nor* and *rin* had only 173 and 392, respectively (Supplementary Table 5). These results suggest that *Cnr* fruit have different gene expression profiles from WT throughout fruit growth and maturation, even before ripening begins.

### Transcriptional Misregulation in the Ripening Mutants Leads to Inhibition or Delay of Molecular Processes

We determined DEGs (*P*_adj_ ≤ 0.05) from the MG and RR stages to identify specific molecular functions altered in *Cnr*, *nor*, and *rin* fruit. First, we compared the ripening mutants to the WT at each stage and obtained a total of 16,085 mutation-related DEGs across all comparisons (Figure 2B). Like the PCA suggested (Figure 2A), *Cnr* MG fruit presented the largest amount of mutation-related DEGs (5,482), while *nor* and *rin* MG had considerably fewer DEGs when compared to the WT counterpart (580 and 269 DEGs, respectively). At the RR stage, large differences between each mutant and WT were observed, with *Cnr* RR fruit displaying once again the largest differences in the amount of mutation-related DEGs (10,582, Figure 2B). The large number of mutation-related DEGs shown by *Cnr* fruit further supports our hypothesis that the *Cnr* mutation more broadly affects fruit development and that *nor* and *rin* appear to be more ripening-specific mutations.

We examined molecular functions based on KEGG annotations that were significantly (*P*_adj_ ≤ 0.05) enriched among the mutation-related DEGs for each *Cnr, nor*, and *rin* fruit at MG and RR (Supplementary Figure 3). Large differences in enriched functions were detected in the *Cnr* MG fruit, which mainly corresponded to alterations in carbohydrate and amino acid metabolism, chlorophyll, and carotenoid biosynthesis, and interestingly many processes related to DNA replication and repair. The lack of green color in *Cnr* MG fruit could be explained by lower expression of photosynthesis and carbon fixation genes. The *nor* MG and *rin* MG fruit showed few alterations compared to WT and were mainly noted in amino acid metabolism and plant hormone signal transduction. In contrast, at the RR stage, the three ripening mutants showed significant alterations across multiple molecular pathways that range from primary and secondary metabolism to transcription, translation, and signaling processes.

We proceeded to mine the mutation-related DEGs for key genes known to affect the fruit traits evaluated in the ripening mutants: color, firmness, TSS, and acidity. We selected five carotenoid biosynthesis genes involved in fruit pigmentation, six genes encoding cell wall degrading enzymes (CWDEs) that promote fruit softening, four genes related to sugar accumulation and transport that impact the fruit’s TSS, and one gene that regulates the levels of citric acid then affecting the fruit’s acidity (Table 1). At the MG stage, we observed that *Cnr* fruit showed significantly lower expression than WT for several of these key genes, consistent with our phenotypic data (Figure 1), including firmness related enzymes and carotenoid biosynthesis genes. MG fruit from the three ripening mutants showed significantly lower gene expression in an important invertase in fruit (*SlSUCR*), which may contribute to the lower levels of TSS observed in all the mutants (Table 1; Klann et al., 1993). At the RR stage, most of the fruit trait-associated genes surveyed in the ripening mutants had a significantly lower expression than WT, in support of the phenotypic data and reinforced by the numerous functional enrichments among the mutation-related DEGs (Supplementary Figure 3). The critical carotenoid biosynthesis gene that encodes *PHYTOENE SYNTHASE 1* (*PSY1)* was significantly lower expressed than WT in the mutant fruit across all stages, accounting for the lack of red pigmentation at the RR stage. Also, downstream genes in the pathway encoding Lycopene β-cyclases (*SlLCY1 and SlLCY2*) were highly expressed in the mutants at the RR stage, suggesting that not only was less lycopene being produced but more was being metabolized. CWDEs were negatively affected across all genotypes, with *Cnr* having the most mutation-related DEGs in this category.

**TABLE 1:** Differential expression of key genes associated with tomato fruit traits in the single ripening mutants *Cnr*, *nor*, and *rin*.

			Mutation comparison						Ripening comparison			
			(Log_2_FC mutant/WT)						(Log_2_FC RR/MG)			
				
Fruit trait	Gene accession	Gene name	*Cnr* MG	*nor* MG	*rin* MG	*Cnr* RR	*nor* RR	*rin* RR	WT	*Cnr*	*nor*	*rin*
Color	*Solyc03g031860*	Phytoene synthase 1 (*SlPSY1*)	–2.41	–1.93	–1.81	–4.08	–3.97	–5.33	3.32	1.65	1.29	
	*Solyc03g123760*	Phytoene desaturase (*SlPDS1*)	–0.63			–0.70						
	*Solyc01g097810*	ζ-carotene desaturase (*SlZDS*)	–0.81			–1.45	–1.19	–1.02	0.96			
	*Solyc04g040190*	Lycopene β-cyclase (*SlLCY1*)	–0.76				2.15	1.94	–2.02	–1.19		
	*Solyc10g079480*	Lycopene β-cyclase (*SlLCY2*)				4.07	4.15	4.48	–3.73			

Firmness	*Solyc10g080210*	Polygalacturonase (*SlPG2A*)	–3.39	–4.40	–3.58	–5.03	–11.38	–8.33	7.54	5.90		2.80
	*Solyc03g111690*	Pectate lyase (*SlPL*)				–2.23	–4.14	–4.64	3.05	1.42		
	*Solyc12g008840*	β-galactosidase 4 (*TBG4*)				–1.97	–2.89	–1.87	1.71			
	*Solyc07g064170*	Pectin methylesterase 1 (*SlPME1*)	–8.27			–8.66		–1.02	–1.21	–1.60		–2.41
	*Solyc07g064180*	Pectin methylesterase 2 (*SlPME2*)	–5.53			–7.65				–2.60		–1.38
	*Solyc01g008710*	Mannan endo-1,4-β-mannosidase (*SlMAN*)	–9.69	–9.11	–4.11	–7.63	–5.66					3.85

Total soluble solids	*Solyc03g083910*	Sucrose accumulator (*SlSUCR*)	–2.50	–2.33	–2.27	–3.88	–5.57	–5.85	2.52			
	*Solyc11g017010*	Sucrose transporter (*SlSUT1*)	1.59			3.67				1.76		
	*Solyc05g007190*	Sucrose transporter (*SlSUT2*)				1.48				1.08		
	*Solyc04g076960*	Sucrose transporter (*SlSUT4*)	0.93			2.27	0.86	2.01	–1.29			0.62

Acidity	*Solyc12g005860*	Aconitate hydratase (*SlACO*)				–0.58	–1.71	–1.55	1.38	1.14		

*Two comparisons were performed: one to capture differences between mutant vs. wild type (WT) fruit at the mature green (MG) and red ripe (RR) stages, and the other to detect differences across ripening (RR vs. MG) in the WT and mutant fruit. Only significant fold changes (Log_2_FC, *P_adj_* ≤ 0.05) are presented.*

We were interested in examining if the *Cnr*, *nor*, and *rin* mutant fruit displayed altered ripening progression or if they were completely inhibited or delayed in ripening events. We performed another set of differential expression analyses comparing RR against MG fruit for WT and each of the mutants to reveal ripening-related DEGs. As anticipated, WT had the largest number of ripening-related DEGs (9,825), while *nor* showed almost no change between the two ripening stages with only 89 DEGs detected (Figure 2C). *Cnr* and *rin* had fewer ripening-related DEGs compared to WT but still exhibited significant changes during the transition between stages with 5,788 and 2,799 DEGs, respectively. Although *Cnr* showed the most differences from WT in mutation-related DEGs (Figure 2B), it had the largest number of ripening-related DEGs (2,454) in common with WT fruit (Figure 2C). *Cnr* also displayed similar functional enrichments (*P*_adj_ ≤ 0.05) to WT among their respective ripening-related DEGs, including photosynthesis-related pathways, carbohydrate, and amino acid metabolism, and plant hormone signal transduction (Supplementary Figure 4). Compared to *Cnr*, *rin* shared a smaller number of ripening-related DEGs (722) and functional enrichments with WT fruit (Figure 2C and Supplementary Figure 4). The number of ripening-related DEGs shared between *nor* and WT fruit was negligent, and no functional enrichments were detected in this set of DEGs.

Similar to our previous analysis, we mined the ripening-related DEGs to determine the patterns of expression of key genes involved in fruit quality traits (Table 1). We observed that *Cnr* and WT showed similar gene expression of *SlPSY1*, *SlLCY1, POLYGALACTURONASE 2A* (*SlPG2A*), pectate lyase (*SlPL*), *PECTIN METHYLESTERASE 1* (*SlPME1*), and *ACTINATE HYDRATASE* (*SlACO*). Fruit from *nor* and *rin* did not have similar ripening expression patterns to WT fruit for those genes, except for the *SlPG2A* and *SlPME1* in *rin*. Altogether, these data indicate that *Cnr* fruit undergo the most similar ripening progression to WT fruit, while *nor* and *rin* fruit have moderate to minimal changes between the MG and RR stages.

### Ripening Mutants Present Alterations in Hormone Networks

Alterations in transcriptional and hormone control likely cause the extensive gene expression differences that lead to the pleiotropic ripening defects in the mutants. Our transcriptional data pointed out that both mutation-related and ripening-related DEGs were significantly enriched (*P*_adj_ ≤ 0.05) in functions related to hormone regulation (Supplementary Figures 3, 4). Thus, we decided to look closer at defects in hormone biosynthesis and signaling in the mutant fruit, with a particular focus on ethylene and ABA as they are known to promote tomato ripening (Zhang et al., 2009; Kumar et al., 2014; Mou et al., 2016).

**FIGURE 3:**
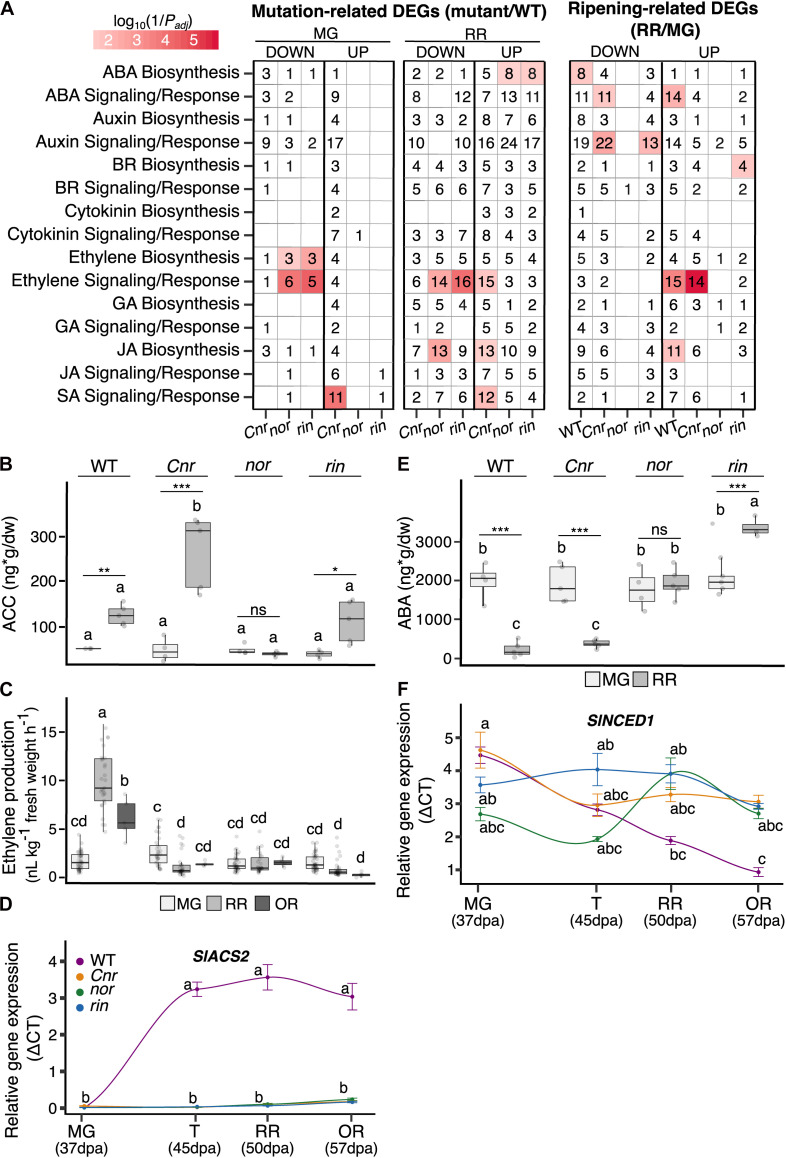
Plant hormone networks altered in the single ripening mutants *Cnr*, *nor*, and *rin*. **(A)** Functional enrichments in hormone functions among differentially expressed genes (DEGs; *P*_adj_ ≤ 0.05) in two comparisons: mutation-related DEGs obtained when comparing each mutant to the wild type (WT) at the mature green (MG) and red ripe (RR) stages, and ripening-related DEGs when the RR stage was compared against MG for each genotype. Each comparison is separated into significant down- and up-regulated DEGs. The heat map colors indicate the significance of the functional enrichment using a log_10_(1/*P*_adj_) scale. Numbers in each tile indicate the number of DEGs within each category. Only significant (*P_adj_* ≤ 0.05) functional enrichments are shown. Hormone measurements of the **(B)** ethylene precursor 1-aminocyclopropane-1-carboxylate (ACC) (*n* = 4–6), **(C)** ethylene (*n* = 5–45), and **(E)** abscisic acid (ABA) (*n* = 4–6) for WT, *Cnr*, *nor*, and *rin* fruit at the MG, RR, and/or overripe (OR) stages. Relative gene expression by RT-qPCR of key hormone biosynthesis genes of **(D)** ethylene and **(F)** ABA across (*n* = 6) across four ripening stages MG, turning (T), RR, and OR for each genotype. Error bars represent standard error between biological replicates of each sample. Letters in **(B–F)** indicate significant differences among genotypes and ripening stages calculated by ANOVA and Tukey HSD (*P* ≤ 0.05). Asterisks in **(B,C,E)** denote significant differences (*P ≤ 0.05, **P ≤ 0.01, ***P ≤ 0.001) between two ripening stages within a single genotype calculated by Student’s *t-*test.

It has been reported multiple times that ethylene production is negatively affected in the *Cnr*, *nor*, and *rin* mutants (Giovannoni, 2007; Liu et al., 2015; Li et al., 2019a). We confirmed that the three ripening mutants do not present the ethylene burst associated with climacteric fruit ripening at any of the stages evaluated, MG, RR, and OR (Figure 3). However, in a one-way ANOVA and Tukey HSD test comparing all genotypes at the MG stage we noted that *Cnr* fruit produced significantly (*P* = 0.0004) more ethylene at the MG stage than WT MG fruit and the other mutants at the equivalent stages. We validated these results with field-grown tomatoes across four field seasons. The results from each season can be found in Supplementary Table 2. To give a sense if ethylene was inhibited at an early step in biosynthesis in the mutants, we measured the accumulation of the immediate ethylene precursor ACC at the MG and RR stages. ACC accumulates typically at the RR stage in WT fruit, reflecting the increase in ethylene biosynthesis and ethylene production. Surprisingly, ACC concentrations also increased in *Cnr* and *rin* fruit during ripening, reaching values similar to WT fruit (Figure 3B); yet the fruit did not produce normal ethylene levels. Moreover, the ACC accumulation in *Cnr* RR fruit was the highest across all genotypes and ripening stages, significantly more than WT RR fruit. These results suggest that the low levels of ethylene in *Cnr* and *rin* RR fruit may be partially explained by inhibition of the final enzymatic step in ethylene biosynthesis.

We found ethylene biosynthesis significantly enriched (*P*_adj_ ≤ 0.05) among mutation-related and ripening-related DEGs in several of the mutants (Figure 3A). At the MG stage, *nor* and *rin* fruit had significantly lower expression of the primary ripening ACC synthases (*SlACS2* and *SlACS4*) and ACC oxidases (*SlACO1* and *SlACO4*). At RR, this pattern was maintained except for *SlACO4*, which was higher than WT for both mutants. *SlACS2* was significantly down-regulated across all mutants and stages compared to WT. We validated the expression patterns of *SlACS2* by RT-qPCR experiment using independent samples from WT and the mutant fruit obtained from another field season (Figure 3C). We included fruit at the T and OR stages in the validation experiment to capture the gene expression dynamics across fruit ripening and senescence.

In *Cnr* MG fruit, *SlACS4* was significantly lower expressed than WT, like the other mutants, but *SlACS2* showed no significant difference. Interestingly, *Cnr* MG fruit had higher gene expression of four ACC oxidases than WT MG fruit, including *SlACO3*, which is involved in System 1 of ethylene biosynthesis. The increased ACC oxidase expression in *Cnr* MG fruit could explain the high ethylene levels detected in these fruit (Figure 3B). At RR, four *ACO* genes had significantly higher expression than WT, except for *SlACO1* that showed no significant differences in RT-qPCR relative expression shown in Supplementary Table 6.

Ethylene signaling and response genes, including *ETHYLENE INSENSITIVE 3* (*EIN3*) and *EIN3-BINDING F-BOX* (*EBF*) homologs, were generally higher expressed in *Cnr* than WT at MG and RR stages. *Nor* and *rin* displayed the opposite trend, with generally lower expression than WT at both stages in these genes (Supplementary Table 4). These patterns were also reflected in significant enrichments (*P*_adj_ ≤ 0.05) of ethylene signaling and response genes at the RR stage (Figure 3). Interestingly, ethylene receptor encoding genes (ETRs) were lower expressed across all genotypes and stages compared to WT. In contrast, ethylene response TFs (ERFs) were generally higher expressed in all genotypes at the RR stage.

We measured ABA levels present in the WT and mutant fruit at the MG and RR stages. A decrease of ABA during ripening was found in WT, consistent with previous reports (Mou et al., 2016). This pattern was also present in *Cnr* fruit. However, in *nor* fruit, ABA remained at the same level across both stages, and *rin* showed a significant increase at the RR stage. ABA biosynthesis was significantly enriched among mutation-related DEGs in *nor* and *rin* RR fruit, consistent with the high ABA levels observed (Figure 3A). We looked at specific ABA biosynthesis genes enriched in *nor* and *rin* that were also down-regulated in WT at the RR stage and found *SlNCED1*, encoding the 9-*cis*-epoxycarotenoid dioxygenase that catalyzes the rate-limiting step in ABA biosynthesis (Ji et al., 2014). We validated the expression of *SlNCED1* with independent samples and additional stages (T and OR) using RT-qPCR (Figure 3C). We also confirmed the expression of an upstream biosynthesis gene, *SlZEP*, encoding a zeaxanthin epoxidase, which was also significantly up-regulated in *nor* at the RR stage and in *rin* at the T stage (Supplementary Table 6). While *Cnr* accumulated ABA, signaling and response genes were altered in at MG and RR fruit, including higher expression in ABRE-binding protein (AREB)/ABRE binding factors (ABFs) at both stages compared to WT. *Nor* and *rin* showed alterations in signaling and response at the RR stage, such as lower expression of receptor protein (PYR/PYL) genes and higher expression of the PP2C phosphatase (Supplementary Table 4).

We observed changes in biosynthesis and signaling of other plant hormones implicated in fruit development, such as auxins, cytokinins, jasmonic acid, and brassinosteroids (Figure 3A). *Cnr* MG fruit had alterations in all hormone pathways examined, further supporting the differences present in *Cnr* phenotype before ripening begins. At the RR stage, all mutants presented multiple defects in hormone metabolism compared to WT. Ripening-related DEGs with hormone functions displayed a similar expression pattern in WT and *Cnr* fruit, whereas *nor* and *rin* displayed low numbers of ripening-related DEGs from these categories.

### Ripening Mutations Influence the Expression Dynamics of *CNR, NOR*, and *RIN* in Fruit

**FIGURE 4:**
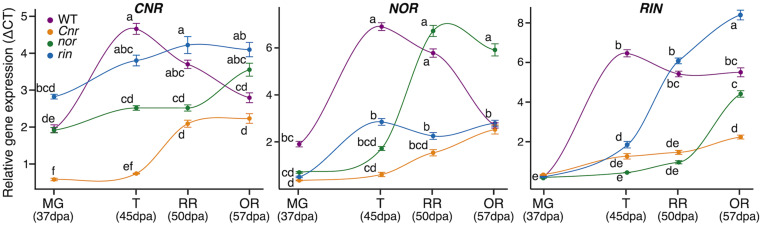
Impact of ripening mutations on the *CNR*, *NOR*, and *RIN* transcription factors. Relative gene expression of the three transcription factors across ripening in the wild type (WT), *Cnr*, *nor*, and *rin* genotypes measured by quantitative reverse transcription (qRT)-PCR. The measurements were done with fruit (*n* = 6) collected at 37 days post anthesis (dpa), equivalent to the mature green stage (MG), 45 dpa, equivalent to the turning stage (T), 50 dpa, equivalent to the red ripe stage (RR), and 57 dpa, equivalent to the overripe (OR) stage. Error bars represent standard error between biological replicates of each sample. Letters indicate significant differences among genotypes and ripening stages calculated by ANOVA and Tukey HSD (*P* ≤ 0.05).

Another way in which the mutations in the *CNR*, *RIN*, and *NOR* may affect gene expression of ripening processes is through direct or indirect interactions with each other. We performed RT-qPCR on fruit from the MG, T, RR, and OR stages in each genotype for each of the genes encoding the ripening TFs (Figure 4). In WT, each TF follows a ripening pattern, peaking in expression at the T stage. Mutations in any of the three TFs led to a decrease or delay in the expression of the other TFs compared to WT. For example, *RIN* expression does not begin to show an increase until the OR stage for *nor* and *Cnr*. A similar pattern was exhibited in *CNR* expression for *nor* and *rin* and *NOR* expression in *rin* and *Cnr*. The *Cnr* fruit displayed the most dramatic decreases in expression across the TFs, while the *nor* fruit showed the most delays.

### Phenotypic Differences in Double Mutants Reveal Genetic Relationships

**FIGURE 5:**
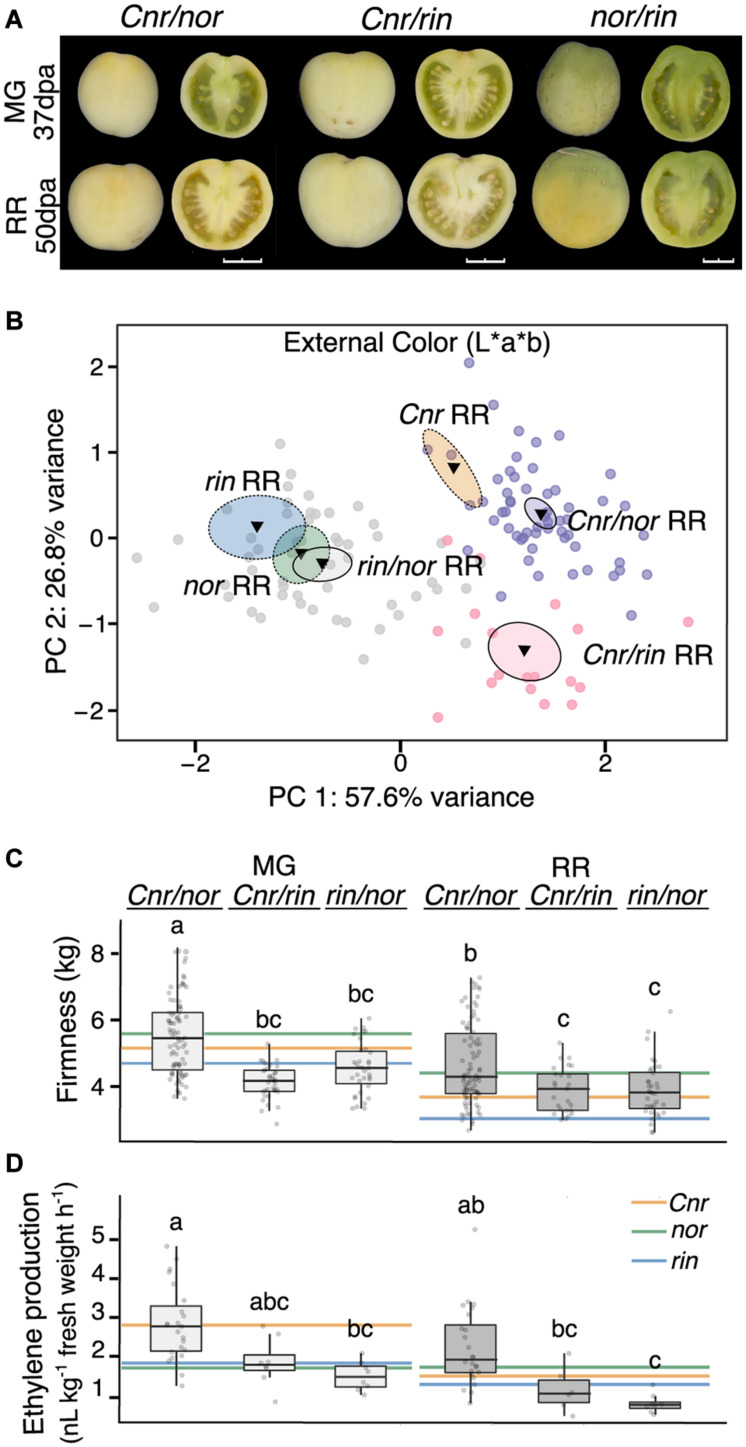
Fruit traits of the homozygous double ripening mutants. **(A)** Homozygous double mutants pictured at the mature green (MG) and red ripe (RR) stages. Fruit shown whole at left and in longitudinal sections at right. Images were extracted and processed with the VideometerLab instrument. Bar represents 2 cm. **(B)** Principal component analysis of fruit external color (*n* = 18–54). The center of gravity is represented by a triangle with surrounding ellipses indicating 95% confidence interval. Dashed ellipses indicate the values of the single mutant parents. **(C)** Texture analysis of fruit firmness at MG and RR stages (*n* = 25–86). **(D)** Ethylene production of MG and RR fruit (*n* = 6-24). Letters indicate significant differences among genotypes and stages (*P* ≤ 0.05). Colored lines indicate averages of the parents at each stage for comparison.

The changes in gene expression of *CNR*, *NOR*, and *RIN* in the ripening mutants indicate that the genes are interconnected during fruit development. In addition, *Cnr* consistently showed earlier defects in fruit traits, gene expression, and hormone pathways. To characterize the combined genetic effects of the mutations on tomato fruit, we generated homozygous double mutants through reciprocal crosses of the single mutants. We then phenotyped the double mutants for fruit traits and ethylene production (Figure 5). Because the reciprocal crosses produced fruit indistinguishable from each other, we report them as only one double mutant (Supplementary Table 7 and Supplementary Figure 5). Fruit of *nor/rin* double mutants were almost indistinguishable from both *nor* and *rin* fruit in appearance and external color. Fruit resulting from any cross with *Cnr* as a parent presented similar visual characteristics (Figure 5A). We also performed a PCA of the color measurements to compare the double mutants to their parental lines at the RR stage and confirmed this observation (Figure 5B and Supplementary Table 7). Based on these observations and our earlier phenotypic and transcriptional data, we confirmed that the *Cnr* mutation affects early fruit development. In contrast, the *nor* and *rin* mutations act during fruit ripening.

If defects in *Cnr* occur earlier in fruit development than those caused by *nor* or *rin*, we expected the *Cnr/rin* and *Cnr/nor* double mutants to behave similarly to *Cnr* and display similar phenotypes (Figures 5C,D). *Cnr/rin* fruit were significantly (*P* ≤ 0.05) less firm than either parent at the MG stage but performed most similarly to *Cnr* at the RR stage. *Cnr/nor* fruit was not distinguishable from either parent in firmness at MG but was firmer (*P* ≤ 0.05) than *Cnr* RR fruit. Interestingly, *Cnr/nor* fruit exhibited high ethylene production at the MG stage like the *Cnr* fruit. At the RR stage, *Cnr/nor* showed a less pronounced decrease in ethylene production, resulting in higher hormone levels than either parent. Although some phenotypic differences were detected, we verified that *Cnr/rin* and *Cnr/nor* resembled the *Cnr* parent for most of the fruit traits measured.

If *nor* and *rin* act synergistically during ripening, the *rin/nor* double mutants would have a more extreme phenotype than either on their own. At the MG stage, *rin/nor* fruit firmness was statistically similar to *rin* (*P* ≤ 0.05; Figure 5C) but became an intermediate phenotype at the RR stage. For ethylene, *rin*/*nor* fruit produced less than either parent at both stages, although not significant, suggesting a combined effect of both mutations.

### Double Mutant *Cnr/nor* Shows Gene Expression Unique From Both Parents

**FIGURE 6:**
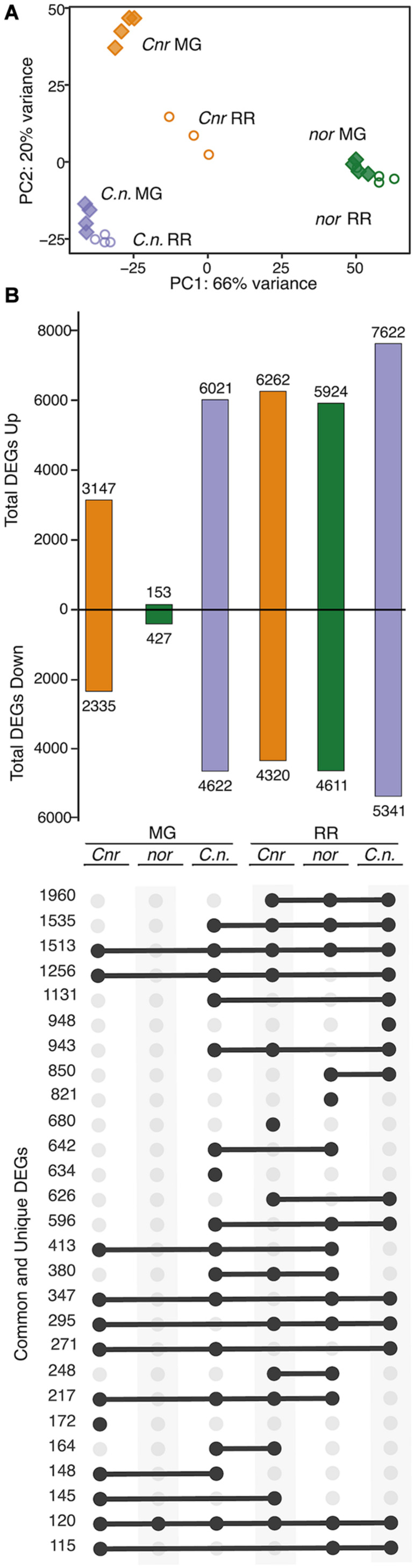
Comparison of *Cnr/nor* double mutant gene expression to parents. **(A)** Principal component analysis of total mapped RNAseq reads for the double mutant *Cnr/nor* (*C.n.*), and the parents *Cnr* and *nor* at the mature green (MG) and red ripe (RR) stages. **(B)** Total, unique, and intersecting differentially expressed genes (DEGs) compared to wild type (WT) for each genotype (mutant/WT) visualized using *UpSetR*. Dots connected by lines indicate common DEGs between categories and single dots indicate unique DEGs, with the numbers of shared or unique genes at the left.

The strong effect of *Cnr* in the double mutant phenotypes led us to investigate if gene expression in the fruit was altered in a similar way. We selected the *Cnr/nor* double mutant to perform an RNAseq experiment of fruit at MG and RR stages and assessed the overall transcriptional changes resulting from the two mutations combined. We conducted a PCA of total mapped reads for MG and RR fruit of *Cnr/nor* and the single mutant parents (Figure 6A). In this analysis, *Cnr/nor* expression appeared more similar to *Cnr* than *nor* in PC1 (66% of variance), but PC2 (20% of variance) accounted for differences between *Cnr* and *Cnr/nor.*

We analyzed mutation-related DEGs (*P*_adj_ ≤ 0.05) in the *Cnr*/*nor* fruit by comparing the gene expression patterns of the double mutant against WT at both MG and RR stages. We then determine which of these mutation-related DEGs were also differentially expressed between the single mutant parents and WT (Figure 6B). Similar to *Cnr*, *Cnr/nor* fruit started with a high number of mutation-related DEGs (10,643) at the MG stage, showing defects in development before the initiation of ripening. However, *Cnr/nor* MG and RR fruit showed more mutation-related DEGs than either *nor* or *Cnr* fruit, including 634 unique DEGs at the MG stage and 948 at the RR stage. These data indicate that the *Cnr/nor* fruit present additional defects than *Cnr* fruit prior to ripening.

Interestingly, *Cnr/nor* and both its parents at the RR stage shared many mutation-related DEGs (1,980) (Figure 6B). These shared mutation-related DEGs were significantly (*P*_adj_ ≤ 0.05) enriched in glycolysis, starch and sucrose metabolism, fructose and mannose metabolism, among others, suggesting that carbohydrate metabolism is altered in all three genotypes (Supplementary Table 4). When we looked again at the key genes associated with fruit traits, we observed greater defects in the double mutant compared to the single mutant parents (Supplementary Table 8).

We only identified 272 ripening-related DEGs (*P*_adj_ ≤ 0.05) in *Cnr*/*nor* fruit, indicating that the double mutant fruit changed very little between the MG and RR stages (Supplementary Table 4). This inhibition of ripening progression is similar to the ripening-related DEG patterns exhibited by *nor* (Figure 2), highlighting a critical difference between *Cnr/nor* and *Cnr*. Overall, the transcriptional data indicate that *Cnr/nor* have stronger alterations in fruit development and more significant inhibition of fruit ripening than the *Cnr* and *nor* fruit.

## Discussion

The spontaneous ripening mutants, *Cnr*, *nor*, and *rin*, are essential genetic tools to untangle the complexity of climacteric fruit ripening (Chen et al., 2020) and to breed for extended shelf-life or field harvest traits in tomato (Kitagawa et al., 2005; Garg et al., 2008). However, thorough phenotyping of the fruit traits affected by these mutants using plants grown under field conditions has been neglected. Here, we produced an extensive quantitative study of fruit quality in the tomato ripening mutants and corroborated it across multiple field seasons. We were able to carefully describe physiological and molecular differences between the mutants by sampling large numbers of fruit and surveying distinct stages through ripening in ways not feasible with greenhouse experiments.

### Delay or Inhibition of Ripening Events Vary in *nor* and *rin*

We determined that some ripening events in the mutants *nor* and *rin* were not completely blocked but severely delayed. By examining the OR stage, we found that the mutation in *nor* may strongly affect firmness and taste while pigment accumulation was only delayed and slightly perturbed (Figure 1). These phenotypes were supported by higher expression of carotenoid biosynthesis genes in *nor* RR than WT and an increase in *SlPSY1* between the MG and RR stages (Table 1 and Supplementary Figure 3). The accumulation of pigments in *nor* fruit, particularly at late stages in development, has gone unnoticed in previous studies, but it partially resembles the CRISPR-NOR mutants (Gao et al., 2019; Wang et al., 2019). In contrast, *rin* fruit showed strong inhibition of pigment accumulation but less dramatic alterations to fruit taste-related traits, only delaying the accumulation of sugars and decrease in acidity (Figure 1). The lack of upregulation of *SlPSY1* in *rin* (Table 1) appears to contribute to the color defects, consistent with evidence that RIN directly regulates this gene (Fujisawa et al., 2013). Both *nor* and *rin* exhibited severe delays or inhibition of ripening-related gene expression changes. While highly similar to WT at the MG stage, *nor* and *rin* fruit showed large deviations from WT at the RR stage (Figure 2B). In fact, the gene expression profiles of *nor* and *rin* RR fruit remained similar to those from WT MG fruit.

The physiological data generated in this study show *nor* and *rin* mutations have different impacts on fruit quality traits. Soluble solids and acid accumulation are negatively impacted in both mutants, but more dramatically in *nor* fruit. In addition, previous reports have demonstrated a similar pattern among volatile profiles of the mutants at the red ripe stage, with *rin* again showing more similarity to WT in flavor related traits (Kovács et al., 2009). This suggests *rin* fruit are less likely to hinder flavor profiles than *nor* fruit when breeding for fresh-market hybrid varieties with extended shelf-life. Although *nor* showed lower quality flavor attributes, its coloration at overripe stages was most similar to WT compared to *rin;* and thus, it can be useful in breeding hybrid varieties when coloration is a critical fruit trait, such as in the case of processing tomato varieties. Overall, this knowledge will provide valuable information on these tradeoffs of using either loci for breeding programs.

Because the *Cnr*, *nor*, and *rin* mutants never acquire equivalent colorations to WT, their ripening stages have been determined based on the fruit’s age expressed as days after anthesis (dpa) or days after the breaker (BR) stage. Sometimes described as BR + 7 days, the RR stage has been the primary developmental time employed for studying the ripening mutants. As we showed here, the OR stage could provide better comparisons against WT RR fruit for mutants with delayed ripening phenotypes. We demonstrated that in the *nor* fruit, the *RIN* and *CNR* genes only begin to increase in expression in a way comparable to WT at the OR stage (Figure 4). This observation corresponds to over a 10-day delay for some of the ripening processes to begin. The delayed ripening events observed in the OR fruit have not been described before in the spontaneous *nor* mutant.

### *Cnr* Is More Than a Ripening Mutant

Although the *Cnr* mutant has been assumed to have normal fruit development before ripening (Lai et al., 2020), there have been indications that the *Cnr* mutant displays defects that are not ripening-specific, such as earlier chlorophyll degradation and altered expression of CWDE (Eriksson et al., 2004; Wang et al., 2020b). We showed that the *Cnr* mutation causes substantial defects in fruit prior to ripening as seen through statistically significant deviations in fruit size, color, firmness, and TA, ethylene production, and gene expression at the MG stage (Supplementary Table 2 and Figures 1–3). Therefore we propose *Cnr* may be more accurately described as a developmental mutant and not exclusively a ripening mutant. Further complementing these results, the *Cnr* fruit displayed large transcriptional deviations from WT that can be traced back as far as 7 dpa (Figure 2 and Supplementary Figure 2). These early development defects are likely a result of reduced *CNR* expression in the mutant, which is typically expressed in locular tissue before fruit maturity (Giovannoni et al., 2017).

Our analysis of ripening-related gene expression in *Cnr* showed striking similarities to WT in the number and functions of genes changing between stages. Moreover, 69.5% of ripening-related DEGs in *Cnr* were shared with WT (Figure 2). These results further support the hypothesis that *Cnr* is not exclusively a ripening mutant. Instead, *Cnr* fruit undergoes gene expression changes consistent with WT “ripening.” However, the ripening-related changes in gene expression that occur in *Cnr* are not enough to compensate for the large defects accumulated in the fruit during growth and maturation. In a recent report, a knockout mutation to the gene body of *CNR* yielded little visible effects on fruit development and ripening (Gao et al., 2019), which suggests that the *Cnr* mutant phenotype may result from more than just a reduced expression of the *CNR* gene as previously reported (Manning et al., 2006). It has also been demonstrated that *Cnr* fruit have genome-wide methylation changes that inhibit ripening-related gene expression (Zhong et al., 2013). The developmental defects observed in *Cnr* are likely caused by these methylation changes, directly or indirectly caused by the *Cnr* mutation (Chen et al., 2018). Thus, to better understand the *Cnr* mutation, more physiological data at earlier stages of development needs to be analyzed and complemented with more in-depth functional analysis of gene expression alterations at the corresponding stages. In addition, further molecular and genetic studies need to be performed and compared against complete *CNR* knockout mutants.

### The *Cnr* Mutant Produces Ethylene Beyond Basal Levels

Previous reports have shown ethylene levels to be very low or even undetectable in the ripening mutants (Giovannoni et al., 2017). Our data support that the mutants never produce a burst in ethylene production, even at the OR stage where more ripening phenotypes are observed (Figure 3B). The orange-red pigmentation in *nor* OR fruit and the similarities of *rin* OR fruit in texture and taste-related attributes to WT RR fruit occur independently of an ethylene burst. These observations evidence that other regulatory mechanisms exist to initiate ripening events outside of ethylene (Li et al., 2019b).

Unlike previous reports, our data consistently showed that *Cnr* presented increased ethylene levels at the MG stage compared to WT (Wang et al., 2020b). Interestingly, *Cnr* fruit produced more of the ethylene precursor ACC than WT at the RR stage. Also, *rin* made equivalent levels to WT fruit. Ethylene biosynthesis is divided into two programs: System 1 produces basal levels of the hormone during development, and System 2 generates the climacteric rise in ethylene during ripening (Mcmurchie et al., 1972). Each of these systems is catalyzed by a different set of ethylene biosynthetic enzymes (Liu et al., 2015). It is clear that all mutants show defects to System 2 of ethylene biosynthesis, but they also appear to have alterations specific to System 1. For example, we observed that *SlACO3*, a System 1-specific ACC oxidase, was higher expressed in *Cnr* fruit than WT (Supplementary Table 6).

### ABA Biosynthesis and Accumulation Is Affected in *nor* and *rin*

The role of ABA in climacteric ripening is not as well explored but has been reported to be complementary to ethylene (Ji et al., 2014). Previous reports in WT fruit have shown that ABA increases until the breaker stage, just before the ethylene burst (Zhang et al., 2009; Mou et al., 2016). ABA has also been shown to induce ethylene production and linked to the NOR transcription factor (Mou et al., 2018). We found that *nor* and *rin* fruit did not show decreases in ABA concentration during ripening like WT did (Figure 3). For *nor*, the constant levels of ABA between MG and RR stages are another example of how fruit ripening events are delayed or inhibited. *RIN* and ABA have been demonstrated to have an inverse relationship where *RIN* expression is repressed with the induction of ABA (Diretto et al., 2020). The significant increase of ABA accumulation in *rin* during ripening suggests that ABA biosynthesis and metabolism are misregulated in this mutant. *rin* fruit appear to present a delayed peak in ABA levels compared to WT fruit. Our results support the indirect interaction between the TFs and ABA during ripening. More developmental stages, genetic manipulations, and exogenous hormone treatments are needed to investigate further the trends of ABA accumulation seen in the ripening mutants.

### *CNR, NOR, and RIN* Act Interdependently

The interactions between the CNR, NOR, and RIN in ripening have been debated in the literature (Chen et al., 2020). The TF RIN directly interacts with *NOR* and *CNR*, binding to their respective promoters, and therefore has been proposed to be the most upstream TF among the three regulators (Fujisawa et al., 2013). Here we provided evidence that the three TFs display at least indirect effects on each other. We have argued that the *Cnr* mutant shows a wide breadth of defects across fruit development before ripening begins, and thus, we propose the *Cnr* mutation is acting before NOR or RIN. This further supports the hypothesis made in Wang et al. (2020b) that *Cnr* acts epistatically to *nor* and *rin*. The gene expression patterns of *CNR*, *NOR*, and *RIN* across ripening stages were decreased or delayed in each of the single ripening mutants. The most substantial variation in gene expression was the downregulation of *NOR* and *RIN* expression across all stages in the *Cnr* mutant (Figure 4).

We present for the first time double ripening mutants, homozygous for both loci, that can be used to see the combined effects of each mutation on fruit development and quality traits. We successfully generated the double mutants by establishing reliable and high throughput genotyping protocols for each mutation and evaluating segregation of the mutant phenotypes in field trials across multiple growing seasons. We obtained double mutants from both reciprocal crosses but saw no fruit phenotypic differences between them, suggesting that the ripening mutations are not influenced by maternal or paternal effects (Supplementary Table 7). Because the *nor* and *rin* mutants look so similar, it was hard to visually determine the individual effects of each mutation on the appearance of *rin/nor* fruit. However, when specific fruit traits were measured, we could detect additive or intermediate fruit phenotypes in this double mutant, supporting the proposed relationship in Wang et al. (2020b; Figure 5). Thus, *nor* and *rin* appear to influence similar fruit traits and act in coordination.

The *Cnr* mutation had a significant effect on the *Cnr/nor* and *Cnr/rin* mutants resulting in fruit with similar appearance and ethylene production to the *Cnr* fruit (Figure 5). When analyzing the gene expression profiles of the *Cnr/nor* fruit, we also observed multiple similarities to the *Cnr* parent, but also several deviations (Figure 6). Surprisingly, *Cnr/nor* was also reminiscent of *nor*, as it displayed few ripening-related gene expression changes, suggesting the inhibition or delay of specific ripening events in *nor* carried over to the double mutant. Here, we proposed that the *Cnr* mutation causes defects throughout fruit development while the *nor* mutation causes defects predominantly in ripening. However, the *Cnr/nor* double mutant showed additional phenotypic and transcriptional defects before ripening than both mutant parents (Figure 6). These observations indicate that in combination with *Cnr*, *nor* may contribute to alterations in early fruit development and the inhibition of ripening progression.

## Conclusion

Our study contributes new information about the spontaneous tomato ripening mutants, which have been employed to study fruit ripening for at least the past two decades. Also, given the importance of both *nor* and *rin* for tomato breeding, the fruit trait data generated in this study could be applied to improve quality in tomato hybrids or at least identify tradeoffs between fruit traits. Ultimately, our results extend knowledge of underlying genetic and molecular factors affecting fruit ripening and quality while providing insights into fruit physiological changes through ripening and senescence.

## Data Availability Statement

The datasets presented in this study can be found in online repositories. The names of the repository/repositories and accession number(s) can be found below: https://www.ncbi.nlm.nih.gov/geo/, GSE163745.

## Author Contributions

BB-U conceived the original research plan. JA and BB-U designed the experiments and generated the double ripening mutant lines. JA established the genotyping strategies for the single and double mutants, performed the fruit trait phenotyping, measured ethylene emissions, extracted RNA, did the qRT-PCRs, and completed other molecular experiments. JA, CS, and PH performed the bioinformatic analyses. JA and CS analyzed and interpreted the data. JA wrote the manuscript with contributions of CS and BB-U. All authors contributed to the article and approved the submitted version.

## Conflict of Interest

The authors declare that the research was conducted in the absence of any commercial or financial relationships that could be construed as a potential conflict of interest.

## Footnotes

1 https://www.aginnovationusa.com

2 http://solgenomics.net

3 https://github.com/vsbuffalo/sam2counts/

## Supplementary Material

Supplementary Figure 1 External color of single ripening mutant fruit at the mature green (MG) stage. Principal component analysis of the external color of wild type (WT), *Cnr*, *rin*, and *nor* fruit measured on the L*a*b* color scale. The center of gravity is represented by a triangle with surrounding ellipses indicating 95% confidence interval.

Supplementary Figure 2 Principal component analysis (PCA) of normalized RNAseq reads for *Cnr*, *nor*, and *rin* wild type fruit at immature and mature green (MG) stages. The RNAseq data of the single mutants at 7 days post anthesis (dpa), 17, 27, and 37 dpa (MG) were obtained from Lü et al. (2018) and reanalyzed using our bioinformatics pipeline.

Supplementary Figure 3 Functional enrichments in Kyoto Encyclopedia of Genes and Genomes (KEGG) functions among differentially expressed genes (DEGs; *P*_adj_ ≤ 0.05). Mutation-related DEGs were obtained by comparing each mutant to the wild type (WT) at the mature green (MG) and red ripe (RR) stages. Each comparison is separated into significant down- and up-regulated DEGs. The heat map colors indicate the significance of the functional enrichment using a log_10_ (1/*P*_adj_) scale. Numbers in each tile indicate the number of DEGs within each category. Only significant (*P_adj_* ≤ 0.05) functional enrichments are shown.

Supplementary Figure 4 Functional enrichments in Kyoto Encyclopedia of Genes and Genomes (KEGG) functions among differentially expressed genes (DEGs; *P*_adj_ ≤ 0.05). Ripening-related DEGs were obtained by comparing the RR stage against MG for each genotype. Each comparison is separated into significant down- and up-regulated DEGs. The heat map colors indicate the significance of the functional enrichment using a log_10_ (1/*P*_adj_) scale. Numbers in each tile indicate the number of DEGs within each category. Only significant (*P_adj_* ≤ 0.05) functional enrichments are shown.

Supplementary Figure 5 Representative fruit from the reciprocal crosses of the double mutants. The maternal genotype is listed first for each double mutant. Fruit are pictured at the mature green (MG) and red ripe (RR) stages. Fruit shown whole at left and in longitudinal sections at right. Images were extracted and processed with the VideometerLab instrument. Bar represents 1.5 cm.

Supplementary Table 1 Primers sequences used for genotyping and RT-qPCRs. Forward (F) and reverse (R) samples are listed for each primer pair.

Supplementary Table 2 Phenotypic data of ripening mutants at the mature green (MG) and red ripe (RR) by field season. Averages, standard deviations (SD), number of biological replicates (N), and statistical differences are listed for each fruit trait measured. Color was measured in the L*a*b* color space. Letters indicate significant differences among both genotypes and stages calculated by an ANOVA and Tukey HSD (*P* ≤ 0.05).

Supplementary Table 3 Summaries of RNAseq read mapping of tomato wild type, single mutant, and double mutant genotypes across immature, mature green (MG), and red ripe (RR) stages. The study each sample originates from is listed under dataset. Immature stages correspond to 7 days post anthesis (dpa), 17, and 27 dpa. Parsed reads are those that passed through quality and adapter trimming.

Supplementary Table 4 Differential expression output from DESeq2 (Love et al., 2014) with functional annotations for the two main comparisons: one to capture differences between mutant vs. wild type (WT) fruit at the mature green (MG) and red ripe (RR) stages, and the other to detect differences across ripening (RR vs. MG) in the WT and double mutant fruit. The RNAseq data of the single mutants at 7 days post anthesis (dpa), 17, 27, and 37 dpa (MG) were obtained from Lü et al. (2018) and reanalyzed using our bioinformatics pipeline. Comparisons are listed in the header of each section. Sub-headers are values returned by the results function in DESeq2. baseMean, the mean of normalized counts of all samples for that gene; log2FoldChange, the logarithm (base 2) of the fold change between each comparison; lfcSE, the standard error of the log2FoldChange; stat, the Wald test statistic for each comparison; *P* = *P*-value generated from the Wald test statistic; *P*adj, adjusted *P*-value as calculated via the Benjamini and Holchberg method. AHRD, Automated Assignment of Human Readable Descriptions; KEGG, Kyoto Encyclopedia of Genes and Genomes.

Supplementary Table 5 Differential expression output from DESeq2 (Love et al., 2014) with functional annotations for the comparisons between mutant vs. wild type (WT) fruit at 7 days post anthesis (dpa), 17, 27, and 37 dpa (MG) were obtained from Lü et al. (2018) and reanalyzed using our bioinformatics pipeline. Comparisons are listed in the header of each section. Sub-headers are values returned by the results function in DESeq2. baseMean, the mean of normalized counts of all samples for that gene; log2FoldChange, the logarithm (base 2) of the fold change between each comparison; lfcSE, the standard error of the log2FoldChange; stat, the Wald test statistic for each comparison; *P*, *P*-value generated from the Wald test statistic; *P*adj, adjusted *P*-value as calculated via the Benjamini and Holchberg method. AHRD, Automated Assignment of Human Readable Descriptions; KEGG, Kyoto Encyclopedia of Genes and Genomes.

Supplementary Table 6 Relative gene expression by RT-qPCR of hormone biosynthesis genes in wild type and ripening mutant (*Cnr*, *nor*, and *rin*) fruit at the mature green (MG; 37 dpa), turning (T; 45 dpa), red ripe (RR; 50 dpa), and overripe (OR; 57 dpa) stages. The tomato *SlUBQ* gene was used as reference gene.

Supplementary Table 7 Fruit phenotypic data of the double mutants obtained by reciprocal crosses. The maternal genotype is listed first in each double mutant genotype. Fruit traits were measured at the mature green (MG) and red ripe (RR) stages. Averages, standard deviations (SD), and number of biological replicates (n) are presented for ethylene emissions and color (measured in the L*a*b* color space).

Supplementary Table 8 Differential expression of key genes associated with tomato fruit traits in the double ripening mutant *Cnr/nor*. Two comparisons were performed: one to capture differences between mutant vs. wild type (WT) fruit at the mature green (MG) and red ripe (RR) stages, and the other to detect differences across ripening (RR vs. MG) in the *Cnr/nor* mutant fruit. Only significant fold changes (Log_2_FC, *P_adj_* ≤ 0.05) are presented.

## References

[B1] Agar I. T. Abak K. Yarsi G. (1994). Effect of Different Maturity Stages on the Keeping Quality of nor (non-ripening), rin (ripening-inhibitor) and Normal Type Tomatoes. *Acta Hortic* 742–753. 10.17660/actahortic.1994.368.88

[B2] Benjamini Y. Hochberg Y. (1995). Controlling the False Discovery Rate: A Practical and Powerful Approach to Multiple Testing. *J. R. Stat. Soc. Ser. B* 57 289–300. 10.1111/j.2517-6161.1995.tb02031.x

[B3] Blanco-Ulate B. Vincenti E. Powell A. L. T. Cantu D. (2013). Tomato transcriptome and mutant analyses suggest a role for plant stress hormones in the interaction between fruit and Botrytis cinerea. *Front. Plant Sci.* 4:142. 10.3389/fpls.2013.00142 23717322 PMC3653111

[B4] Bolger A. M. Lohse M. Usadel B. (2014). Trimmomatic: A flexible trimmer for Illumina sequence data. *Bioinformatics* 2014:170. 10.1093/bioinformatics/btu170 24695404 PMC4103590

[B5] Casteel C. L. De Alwis M. Bak A. Dong H. Whitham S. A. Jander G. (2015). Disruption of ethylene responses by Turnip mosaic virus mediates suppression of plant defense against the green peach aphid vector. *Plant Physiol.* 169 209–218. 10.1104/pp.15.00332 26091820 PMC4577379

[B6] Chen T. Qin G. Tian S. (2020). Regulatory network of fruit ripening: current understanding and future challenges. *New Phytol.* 228 1219–1226. 10.1111/nph.16822 32729147

[B7] Chen W. Yu Z. Kong J. Wang H. Li Y. Zhao M. (2018). Comparative WGBS identifies genes that influence non-ripe phenotype in tomato epimutant Colourless non-ripening. *Sci. China Life Sci.* 61 244–252. 10.1007/s11427-017-9206-5 29288427

[B8] Conway J. R. Lex A. Gehlenborg N. (2017). UpSetR: An R package for the visualization of intersecting sets and their properties. *Bioinformatics* 2017:364. 10.1093/bioinformatics/btx364 28645171 PMC5870712

[B9] De F. Maintainer M. De Mendiburu F. (2017). *Package “agricolae” Title Statistical Procedures for Agricultural Research. Stat. Proced. Agric. Res. Version 1.3-3.*

[B10] Diretto G. Frusciante S. Fabbri C. Schauer N. Busta L. Wang Z. (2020). Manipulation of β-carotene levels in tomato fruits results in increased ABA content and extended shelf life. *Plant Biotechnol. J.* 18 1185–1199. 10.1111/pbi.13283 31646753 PMC7152610

[B11] Eriksson E. M. Bovy A. Manning K. Harrison L. Andrews J. De Silva J. (2004). Effect of the Colorless non-ripening Mutation on Cell Wall Biochemistry and Gene Expression during Tomato Fruit Development and Ripening 1[w]. *Am. Soc. Plant Biol.* 136 4184–4197. 10.1104/pp.104.045765 15563627 PMC535848

[B12] Fujisawa M. Nakano T. Shima Y. Ito Y. (2013). A large-scale identification of direct targets of the tomato MADS box transcription factor RIPENING INHIBITOR reveals the regulation of fruit ripening. *Plant Cell* 25 371–386. 10.1105/tpc.112.108118 23386264 PMC3608766

[B13] Gao Y. Wei W. Fan Z. Zhao X. Zhang Y. Jing Y. (2020). Re-evaluation of the nor mutation and the role of the NAC-NOR transcription factor in tomato fruit ripening. *J. Exp. Bot* 2020:131. 10.1093/jxb/eraa131 32338291 PMC7307841

[B14] Gao Y. Zhu N. Zhu X. Wu M. Jiang C.-Z. Grierson D. (2019). Diversity and redundancy of the ripening regulatory networks revealed by the fruitENCODE and the new CRISPR/Cas9 CNR and NOR mutants. *Hortic. Res.* 6:39. 10.1038/s41438-019-0122-x 30774962 PMC6370854

[B15] Garg N. Cheema D. S. Dhatt A. S. (2008). Utilization of rin, nor, and alc alleles to extend tomato fruit availability. *Int. J. Veg. Sci.* 14 41–54. 10.1080/19315260801890526

[B16] Giovannoni J. J. (2007). Fruit ripening mutants yield insights into ripening control. *Curr. Opin. Plant Biol.* 10 283–289. 10.1016/j.pbi.2007.04.008 17442612

[B17] Giovannoni J. Nguyen C. Ampofo B. Zhong S. Fei Z. (2017). The Epigenome and Transcriptional Dynamics of Fruit Ripening. *Annu. Rev. Plant Biol.* 68 61-84. 10.1146/annurev-arplant-042916 28226232

[B18] Giovannoni J. Tanksley S. Vrebalov J. Noensie F. (2004). *NOR gene composition and methods for use thereof.* U.S. Patent No US 6,762,347,B1. Washington, DC: U.S. Patent and Trademark

[B19] Ito Y. Nishizawa-Yokoi A. Endo M. Mikami M. Shima Y. Nakamura N. (2017). Re-evaluation of the rin mutation and the role of RIN in the induction of tomato ripening. *Nat. Plants* 1:5. 10.1038/s41477-017-0041-5 29085071

[B20] Ji K. Kai W. Zhao B. Sun Y. Yuan B. Dai S. (2014). SlNCED1 and SlCYP707A2: Key genes involved in ABA metabolism during tomato fruit ripening. *J. Exp. Bot.* 65 5243–5255. 10.1093/jxb/eru288 25039074 PMC4157709

[B21] Karlova R. Chapman N. David K. Angenent G. C. Seymour G. B. De Maagd R. A. (2014). Transcriptional control of fleshy fruit development and ripening. *J. Exp. Bot.* 65 4527–4541. 10.1093/jxb/eru316 25080453

[B22] Kassambara A. Mundt F. Kassambara A. Mundt F. (2017). Factoextra: extract and visualize the results of multivariate data analyses. *R. Packag. Version* 1 337–354.

[B23] Kitagawa M. Ito H. Shiina T. Nakamura N. Inakuma T. Kasumi T. (2005). Characterization of tomato fruit ripening and analysis of gene expression in F1 hybrids of the ripening inhibitor (rin) mutant. *Physiol. Plant.* 123 331–338. 10.1111/j.1399-3054.2005.00460.x

[B24] Klann E. M. Chetelat R. T. Bennett A. B. (1993). Expression of Acid Invertase Gene Controls Sugar Composition in Tomato (Lycopersicon) Fruit. *Plant Physiol.* 103 863–870. 10.1104/pp.103.3.863 12231984 PMC159057

[B25] Kopeliovitch E. Rabinowitch H. D. Mizrahi Y. Kedar N. (1979). The potential of ripening mutants for extending the storage life of the tomato fruit. *Euphytica* 1979:BF00029179. 10.1007/BF00029179

[B26] Kovács K. Fray R. G. Tikunov Y. Graham N. Bradley G. Seymour G. B. (2009). Effect of tomato pleiotropic ripening mutations on flavour volatile biosynthesis. *Phytochemistry* 70 1003–1008. 10.1016/j.phytochem.2009.05.014 19539963

[B27] Kumar R. Khurana A. Sharma A. K. (2014). Role of plant hormones and their interplay in development and ripening of fleshy fruits. *J. Exp. Bot.* 65 4561–4575. 10.1093/jxb/eru277 25028558

[B28] Lai T. Wang X. Ye B. Jin M. Chen W. Wang Y. (2020). Molecular and functional characterization of the SBP-box transcription factor SPL-CNR in tomato fruit ripening and cell death. *J. Exp. Bot.* 71 2995–3011. 10.1093/jxb/eraa067 32016417 PMC7260717

[B29] Langmead B. Salzberg S. L. (2012). Fast gapped-read alignment with Bowtie 2. *Nat. Methods* 9, 357–359. 22388286 10.1038/nmeth.1923 PMC3322381

[B30] Lê S. Josse J. Husson F. (2008). FactoMineR: An R package for multivariate analysis. *J. Stat. Softw* 2008:01. 10.18637/jss.v025.i01

[B31] Li S. Chen K. Grierson D. (2019a). A critical evaluation of the role of ethylene and MADS transcription factors in the network controlling fleshy fruit ripening. *New Phytol.* 221 1724–1741. 10.1111/nph.15545 30328615

[B32] Li S. Xu H. Ju Z. Cao D. Zhu H. Fu D. (2018). The RIN-MC Fusion of MADS-Box Transcription Factors Has Transcriptional Activity and Modulates Expression of Many Ripening Genes. *Plant Physiol.* 176 891–909. 10.1104/pp.17.01449 29133374 PMC5761797

[B33] Li S. Zhu B. Pirrello J. Xu C. Zhang B. Bouzayen M. (2019b). Roles of RIN and ethylene in tomato fruit ripening and ripening-associated traits. *New Phytol.* 2019:16362. 10.1111/nph.16362 31814125 PMC7154718

[B34] Liu M. Pirrello J. Chervin C. Roustan J.-P. Bouzayen M. (2015). Ethylene Control of Fruit Ripening: Revisiting the Complex Network of Transcriptional Regulation. *Plant Physiol.* 169 2380–2390. 10.1104/pp.15.01361 26511917 PMC4677914

[B35] Love M. I. Huber W. Anders S. (2014). Moderated estimation of fold change and dispersion for RNA-seq data with DESeq2. *Genome Biol.* 15:550. 10.1186/s13059-014-0550-8 25516281 PMC4302049

[B36] Lü P. Yu S. Zhu N. Chen Y.-R. Zhou B. Pan Y. (2018). Genome Encode Analyses Reveal the Basis of Convergent Evolution of Fleshy Fruit. *Ssrn* 2018:3155803. 10.2139/ssrn.3155803 30250279

[B37] Manning K. Tor M. Poole M. Hong Y. Thompson A. J. King G. J. (2006). A naturally occurring epigenetic mutation in a gene encoding an SBP-box transcription factor inhibits tomato fruit ripening. *Nat. Genet.* 38 948–952. 10.1038/ng1841 16832354

[B38] Mcmurchie E. J. Mcglasson W. B. Eaks I. L. (1972). Treatment of fruit with propylene gives information about the biogenesis of ethylene. *Nature* 1972:237235a0. 10.1038/237235a0 4557321

[B39] Moriya Y. Itoh M. Okuda S. Yoshizawa A. C. Kanehisa M. (2007). KAAS: An automatic genome annotation and pathway reconstruction server. *Nucleic Acids Res.* 35 W182–W185. 10.1093/nar/gkm321 17526522 PMC1933193

[B40] Mou W. Li D. Bu J. Jiang Y. Khan Z. U. Luo Z. (2016). Comprehensive Analysis of ABA Effects on Ethylene Biosynthesis and Signaling during Tomato Fruit Ripening. *PLoS One* 11:e0154072. 10.1371/journal.pone.0154072 27100326 PMC4839774

[B41] Mou W. Li D. Luo Z. Li L. Mao L. Ying T. (2018). SlAREB1 transcriptional activation of NOR is involved in abscisic acid-modulated ethylene biosynthesis during tomato fruit ripening. *Plant Sci.* 276 239–249. 10.1016/J.PLANTSCI.2018.07.015 30348324

[B42] Osei M. K. Danquah A. Danquah E. Adu-Dapaah (2017). An overview of tomato fruit-ripening mutants and their use in increasing shelf life of tomato fruits. *Afr. J. Agric. Res.* 12 3520–3528. 10.5897/ajar2017.12756

[B43] Osorio S. Carneiro R. T. Lytovchenko A. McQuinn R. Sørensen I. Vallarino J. G. (2020). Genetic and metabolic effects of ripening mutations and vine detachment on tomato fruit quality. *Plant Biotechnol. J.* 18 106–118. 10.1111/pbi.13176 31131540 PMC6920187

[B44] Robinson R. W. Tomes M. L. (1968). Ripening inhibitor: a gene with múltiple effects on ripening. *Tomato Genet. Coop.* 18 36–37.

[B45] Shinozaki Y. Nicolas P. Fernandez-Pozo N. Ma Q. Evanich D. J. Shi Y. (2018). High-resolution spatiotemporal transcriptome mapping of tomato fruit development and ripening. *Nat. Commun.* 9:364. 10.1038/s41467-017-02782-9 29371663 PMC5785480

[B46] Silva C. J. van den Abeele C. Ortega-Salazar I. Papin V. Adaskaveg J. A. Wang D. (2021). Host susceptibility factors render ripe tomato fruit vulnerable to fungal disease despite active immune responses. *J. Exp. Bot.* 2021:601. 10.1093/jxb/eraa601 33462583 PMC8006553

[B47] Thompson A. J. Tor M. Barry C. S. Vrebalov J. Orfila C. Jarvis M. C. (1999). Molecular and genetic characterization of a novel pleiotropic tomato-ripening mutant. *Plant Physiol.* 120 383–390. 10.1104/pp.120.2.383 10364389 PMC59276

[B48] Tieman D. Zhu G. Resende M. F. R. Lin T. Nguyen C. Bies D. (2017). A chemical genetic roadmap to improved tomato flavor. *Science* 355 391–394. 10.1126/science.aal1556 28126817

[B49] Tigchelaar E. C. Tomes M. L. Kerr E. A. Barman R. J. (1973). A new fruit ripening mutant, non-ripning (nor). *Rep. Tomato Genet. Coop.* 23 33–34.

[B50] Tigchelaar E. McGlasson W. Franklin M. (1978). Natural and ethephon-stimulated ripening of F_1_ hybrids of the ripening inhibitor (*rin*) and non-ripening (*nor*) mutants of tomato (*Lycopevsicon esculentum* Mill.). *Aust. J. Plant Physiol*. 1978:PP9780449

[B51] Wang R. Angenent G. C. Seymour G. de Maagd R. A. (2020a). Revisiting the Role of Master Regulators in Tomato Ripening. *Trends Plant Sci.* 25 291–301. 10.1016/j.tplants.2019.11.005 31926765

[B52] Wang R. da Rocha, Tavano E. Lammers M. Martinelli A. Angenent G. (2019). Re-evaluation of transcription factor function in tomato fruit development and ripening with CRISPR/Cas9-mutagenesis. *Sci. Rep.* 2019 1–10. 10.1038/s41598-018-38170-6 30737425 PMC6368595

[B53] Wang R. Lammers M. Tikunov Y. Bovy A. G. Angenent G. C. de Maagd R. A. (2020b). The rin, nor and Cnr spontaneous mutations inhibit tomato fruit ripening in additive and epistatic manners. *Plant Sci.* 294:110436. 10.1016/j.plantsci.2020.110436 32234221

[B54] Ye J. Coulouris G. Zaretskaya I. Cutcutache I. Rozen S. Madden T. L. (2012). Primer-BLAST: a tool to design target-specific primers for polymerase chain reaction. *BMC Bioinformatics* 2012:134. 10.1186/1471-2105-13-134 22708584 PMC3412702

[B55] Zhang M. Yuan B. Leng P. (2009). The role of ABA in triggering ethylene biosynthesis and ripening of tomato fruit. *J. Exp. Bot.* 60 1579–1588. 10.1093/jxb/erp026 19246595 PMC2671613

[B56] Zhong S. Fei Z. Chen Y.-R. Zheng Y. Huang M. Vrebalov J. (2013). Single-base resolution methylomes of tomato fruit development reveal epigenome modifications associated with ripening. *Nat. Biotechnol.* 31 154–159. 10.1038/nbt.2462 23354102

