# Supplementary material for: Single and Double Mutations in Tomato Ripening Transcription Factors Have Distinct Effects on Fruit Development and Quality Traits
Source: Front Plant Sci. 2021 Apr 27;12:647035. doi: 10.3389/fpls.2021.647035 (PMC8110730; doi:10.3389/fpls.2021.647035)
Supplement: Supplementary Figure 1 — External color of single ripening mutant fruit at the mature green (MG) stage. Principal component analysis of the external color of wild type (WT), Cnr, rin, and nor fruit measured on the L*a*b* color scale. The center of gravity is represented by a triangle with surrounding ellipses indicating 95% confidence interval. [file Data_Sheet_1.docx]

Supplemental Material

# Supplemental Figures and Tables

## Supplemental Tables

**Supplemental Table S1.** Primers sequences used for genotyping and RT-qPCRs. Forward (F) and reverse (R) samples are listed for each primer pair.

| **Primer Pair** | **Accession** | **Type** | **Sequence** | **Reference** |
| --- | --- | --- | --- | --- |
| *SlCNR* promoter | *﻿Solyc02g077920* | Genotyping | F- CTAATTTTGTTAGCTACATCACAAATGACAC | This study |
|  |  |  | R- ATATGTGGTTAACTTAACAATCTAGTATAGG |  |
| *SlCNR* promoter  (bisulfite treated) | *﻿Solyc02g077920* | Genotyping | F- GTAAYTAATTTTGTTAGYTAYATYAYAAATGATAT | This study |
|  |  |  | R- RTRRTTAACTTAACAATCTARTATAAA |  |
| *SlNOR* | *﻿Solyc10g006880* | Genotyping | F- AGCCGAAAGAGGTTCGACATAGA | This study |
|  |  |  | R- CGATCCCAACATATCATGCAAATCA |  |
| *RIN* (wild type allele) | *Solyc05g012017* | Genotyping | F- TAGACATGAACAGCCTTCTC | By correspondence with Dr. Yashiro Ito |
|  |  |  | R- GGTTAGCTAATTAAACTATC |  |
| *rin* (mutant allele) | *Solyc05g012017* | Genotyping | F- TAGACATGAACAGCCTTCTC | By correspondence with Dr. Yashiro Ito |
|  |  |  | R- GGTACAACTCCAGTAGCATC |  |
| *SlCNR* | *﻿Solyc02g077920* | RT-qPCR | F- TTCCCGGATTTCTAAGCAAATTGT | Karlova et al. 2013 |
|  |  |  | R- GTTGGAATGTCAACATGGATATGCA |  |
| *SlNOR* | *﻿Solyc10g006880* | RT-qPCR | F- AGAGAACGATGCATGGAGGTTTGT | Yu et. al. 2018 |
|  |  |  | R- CTGGCTCAGGAAATTGGCAATGG |  |
| *SlRIN* | *Solyc05g012017* | RT-qPCR | F- GCAGCAATTCAAGTATGTCCAA | This study |
|  |  |  | R- CCCAAATCCTCACCTAGCAA |  |
| *SlACS2* | *Solyc01g095080* | RT-qPCR | F- TTGTTATGGCTGGTGGTGCC | This study |
|  |  |  | R- TGCTGGGTAGTATGGTGAAGGT |  |
| *SlACO1* | *Solyc07g049530* | RT-qPCR | F- ACAAACAGACGGGACACGAA | Liu et al. 2015 |
|  |  |  | R- CTCTTTGGCTTGAAACTTGA |  |
| *SlNCED1* | *Solyc07g056570* | RT-qPCR | F- TGGGCTCTTCGGACTTGTTG | Blanco-Ulate et al. 2013 |
|  |  |  | R- TTTAAGATCGCCGGTGGGTG |  |
| *SlZEP* | *Solyc02g090890* | RT-qPCR | F- TGATGATGCTTTAGAGCGTG | Duan et al. 2012 |
|  |  |  | R- TATGTGAGATGGACCCGAC |  |
| *SlACO3* | *Solyc07g049550* | RT-qPCR | F- CAAGCAAGTTTATCCGAAAT | Liu et al. 2015 |
|  |  |  | R- CATTAGCTTCCATAGCCTTC |  |

| **Genotype** | **Stage** | **Relative**  **Expression** | ***SlACO1*** | ***SlACO3*** | ***SlZEP*** |
| --- | --- | --- | --- | --- | --- |
| Wild type | MG | Average | 17.672 | 0.156 | 1.101 |
|  |  | SD | 4.831 | 0.060 | 0.272 |
|  | T | Average | 45.539 | 0.092 | 0.645 |
|  |  | SD | 5.569 | 0.018 | 0.151 |
|  | RR | Average | 35.712 | 0.087 | 0.671 |
|  |  | SD | 2.169 | 0.018 | 0.143 |
|  | OR | Average | 21.241 | 0.206 | 0.508 |
|  |  | SD | 6.293 | 0.113 | 0.070 |
| *Cnr* | MG | Average | 21.646 | 4.919 | 0.640 |
|  |  | SD | 14.461 | 0.716 | 0.137 |
|  | T | Average | 16.174 | 3.653 | 0.516 |
|  |  | SD | 8.477 | 0.703 | 0.098 |
|  | RR | Average | 9.286 | 2.453 | 0.502 |
|  |  | SD | 2.364 | 0.318 | 0.051 |
|  | OR | Average | 4.272 | 2.567 | 0.512 |
|  |  | SD | 2.306 | 0.781 | 0.079 |
| *nor* | MG | Average | 21.727 | 0.130 | 1.366 |
|  |  | SD | 3.126 | 0.009 | 1.145 |
|  | T | Average | 26.898 | 0.168 | 1.121 |
|  |  | SD | 13.958 | 0.041 | 0.214 |
|  | RR | Average | 24.835 | 0.215 | 1.614 |
|  |  | SD | 7.654 | 0.198 | 0.441 |
|  | OR | Average | 14.850 | 0.220 | 1.347 |
|  |  | SD | 4.128 | 0.269 | 0.842 |
| *rin* | MG | Average | 16.390 | 0.277 | 1.049 |
|  |  | SD | 3.265 | 0.046 | 0.233 |
|  | T | Average | 15.482 | 0.945 | 1.358 |
|  |  | SD | 8.536 | 0.390 | 0.465 |
|  | RR | Average | 21.438 | 0.345 | 1.106 |
|  |  | SD | 13.500 | 0.028 | 0.224 |
|  | OR | Average | 13.513 | 0.202 | 1.631 |
|  |  | SD | 3.823 | 0.113 | 0.218 |

**Supplemental Table S6.** Relative gene expression by RT-qPCR of hormone biosynthesis genes in wild type and ripening mutant (*Cnr*, *nor*, and *rin*) fruit at the mature green (MG; 37 dpa), turning (T; 45 dpa), red ripe (RR; 50 dpa), and overripe (OR; 57 dpa) stages. The tomato *SlUbiquitin* gene was used as reference gene.

**Supplemental Table S7.** Fruit phenotypic data of the double mutants obtained by reciprocal crosses. The maternal genotype is listed first in each double mutant genotype. Fruit traits were measured at the mature green (MG) and red ripe (RR) stages. Averages, standard deviations (SD), and number of biological replicates (n) are presented for ethylene emissions and color (measured in the L*a*b* color space).

|  |  | **Ethylene**  **(nL kg^-1^ fresh weight h^-1^)** | | | **Color (L)** | | | **Color (*a)** | | | **Color (*b)** | | |
| --- | --- | --- | --- | --- | --- | --- | --- | --- | --- | --- | --- | --- | --- |
| **Genotype** | **Stage** | **Average** | **SD** | **n** | **Average** | **SD** | **n** | **Average** | **SD** | **n** | **Average** | **SD** | **n** |
| *Cnr/nor* | MG | 3.142 | 0.990 | 12 | 74.981 | 2.630 | 67 | -8.151 | 2.022 | 67 | 25.006 | 3.313 | 67 |
| *nor/Cnr* | MG | 2.514 | 0.776 | 10 | 74.208 | 2.797 | 108 | -8.615 | 1.741 | 108 | 25.111 | 4.164 | 108 |
| *Cnr/nor* | RR | 2.105 | 0.691 | 12 | 76.255 | 2.215 | 71 | -5.063 | 1.233 | 71 | 26.235 | 4.721 | 71 |
| *nor/Cnr* | RR | 2.230 | 1.310 | 12 | 76.868 | 2.647 | 95 | -5.278 | 1.182 | 95 | 27.845 | 5.384 | 95 |
| *Cnr/rin* | MG | 2.248 | 0.285 | 4 | 69.005 | 3.643 | 27 | -7.097 | 1.805 | 27 | 20.189 | 3.935 | 27 |
| *rin/Cnr* | MG | 1.407 | 0.828 | 4 | 76.209 | 1.725 | 22 | -7.741 | 1.334 | 22 | 26.673 | 5.158 | 22 |
| *Cnr/rin* | RR | 1.773 | 0.774 | 3 | 68.017 | 3.038 | 13 | -4.798 | 0.894 | 13 | 19.075 | 3.222 | 13 |
| *rin/Cnr* | RR | 0.663 | 0.278 | 3 | 75.664 | 2.057 | 5 | -5.598 | 1.466 | 5 | 23.532 | 5.668 | 5 |
| *rin/nor* | MG | 1.251 | 0.297 | 4 | 65.786 | 2.994 | 27 | -10.783 | 1.974 | 27 | 25.452 | 3.531 | 27 |
| *nor/rin* | MG | 1.600 | 0.408 | 4 | 66.111 | 3.439 | 36 | -10.613 | 1.783 | 36 | 27.003 | 3.756 | 36 |
| *rin/nor* | RR | 0.811 | 0.309 | 4 | 67.990 | 3.145 | 20 | -7.932 | 1.773 | 20 | 32.669 | 5.164 | 20 |
| *nor/rin* | RR | 0.603 | 0.126 | 4 | 66.465 | 2.961 | 34 | -8.356 | 1.998 | 34 | 33.709 | 4.190 | 34 |

**Supplemental** **Table S8.** Differential expression of key genes associated with tomato fruit traits in the double ripening mutant *Cnr/nor*. Two comparisons were performed: one to capture differences between mutant vs. wild type (WT) fruit at the mature green (MG) and red ripe (RR) stages, and the other to detect differences across ripening (RR vs. MG) in the *Cnr/nor* fruit. Only significant fold changes (Log_2_FC, *P_adj_*≤0.05) are presented.

|  |  |  | **Mutation comparison**  **(log_2_FC mutant/ WT)** | | **Ripening comparison**  **(log_2_FC RR/ MG)** |
| --- | --- | --- | --- | --- | --- |
| **Fruit**  **Trait** | **Gene**  **Accession** | **Gene**  **Name** | ***Cnr/nor***  **MG** | ***Cnr/nor***  **RR** | ***Cnr/nor*** |
| Color | *Solyc03g031860* | Phytoene synthase 1  (*SlPSY1*) | -3.16 | -5.81 |  |
|  | *Solyc03g123760* | Phytoene desaturase  (*SlPDS1*) | -1.34 | -1.54 |  |
|  | *Solyc01g097810* | ζ-carotene desaturase  (*SlZDS*) | -0.67 | -1.58 |  |
|  | *Solyc04g040190* | Lycopene ß-cyclase  (*SlLCYB1*) | -0.81 | 0.80 |  |
|  | *Solyc10g079480* | Lycopene ß-cyclase (*SlLCYB2*) | 1.13 | 4.50 |  |
| Firmness | *Solyc10g080210* | Polygalacturonase (*SlPG2A*) | -3.19 | -10.23 |  |
|  | *Solyc03g111690* | Pectate lyase  (*SlPL*) | -4.16 | -5.44 | 1.76 |
|  | *Solyc12g008840* | ß-galactosidase 4  (*TBG4*) | -1.02 | -3.14 |  |
|  | *Solyc07g064170* | Pectin methylesterase 1  (*SlPME1*) | -10.43 | -8.78 |  |
|  | *Solyc07g064180* | Pectin methylesterase 2  (*SlPME2*) | -7.78 | -7.27 |  |
|  | *Solyc01g008710* | Mannan endo-1,4-ß-mannosidase | -8.33 | -6.85 |  |
| Total Soluble Solids | *Solyc03g083910* | Sucrose accumulator  (*SlSUCR*) | -3.72 | -4.79 |  |
|  | *Solyc11g017010* | Sucrose transporter (*SlSUT1*) | 1.32 | 1.78 |  |
|  | *Solyc05g007190* | Sucrose transporter (*SlSUT2*) | 1.20 | 1.69 |  |
|  | *Solyc04g076960* | Sucrose transporter (*SlSUT4*) | 0.62 | 1.90 |  |
| Acidity | *Solyc12g005860* | Aconitate hydratase  (ACO) |  | -1.12 |  |

## Supplemental Figures

**Supplemental Figure S1.** External color of single ripening mutant fruit at the mature green (MG) stage. Principal component analysis of the external color of wild type (WT), *Cnr*, *rin*, and *nor* fruit measured on the L*a*b* color scale. The center of gravity is represented by a triangle with surrounding ellipses indicating 95% confidence interval.

**Supplemental Figure S2.** Principal component analysis (PCA) of normalized RNAseq reads for *Cnr*, *rin,* *nor*, and wild type fruit at immature and mature green (MG) stages. The RNAseq data of the single mutants at 7 days post anthesis (dpa), 17 dpa, and 27 dpa, and 37 dpa (MG) were obtained from Lü et al (2018) and re-analyzed using our bioinformatics pipeline.

**Supplemental Figure S3.** Functional enrichments in Kyoto Encyclopedia of Genes and Genomes (KEGG) functions among differentially expressed genes (DEGs; *P_adj_* ≤ 0.05). Mutation-related DEGs were obtained by comparing each mutant to the wild type (WT) at the mature green (MG) and red ripe (RR) stages. Each comparison is separated into significant down- and up-regulated DEGs. The heat map colors indicate the significance of the functional enrichment using a log_10_ (1/*P_adj_*) scale. Numbers in each tile indicate the number of DEGs within each category. Only significant (*P_adj_* ≤ 0.05) functional enrichments are shown.

**Supplemental Figure S4**. Functional enrichments in Kyoto Encyclopedia of Genes and Genomes (KEGG) functions among differentially expressed genes (DEGs; *P_adj_* ≤ 0.05). Ripening-related DEGs were obtained by comparing the RR stage against MG for each genotype. Each comparison is separated into significant down- and up-regulated DEGs. The heat map colors indicate the significance of the functional enrichment using a log_10_ (1/*P_adj_*) scale. Numbers in each tile indicate the number of DEGs within each category. Only significant (*P_adj_* ≤ 0.05) functional enrichments are shown.


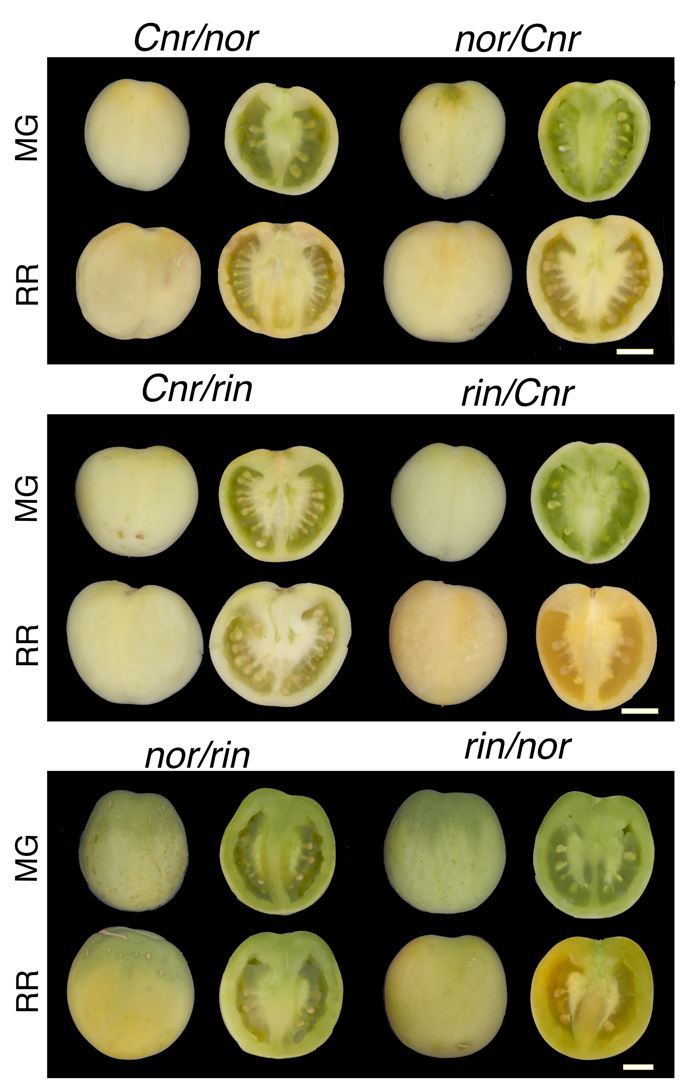


**Supplemental Figure S5.** Representative fruit from the reciprocal crosses of the double mutants. The maternal genotype is listed first for each double mutant. Fruit are pictured at the mature green (MG) and red ripe (RR) stages. Fruit shown whole at left and in longitudinal sections at right. Images were extracted and processed with the VideometerLab instrument. Bar represents 1.5 cm.
